# Eigenspectra optoacoustic tomography achieves quantitative blood oxygenation imaging deep in tissues

**DOI:** 10.1038/ncomms12121

**Published:** 2016-06-30

**Authors:** Stratis Tzoumas, Antonio Nunes, Ivan Olefir, Stefan Stangl, Panagiotis Symvoulidis, Sarah Glasl, Christine Bayer, Gabriele Multhoff, Vasilis Ntziachristos

**Affiliations:** 1 Institute of Biological and Medical Imaging (IBMI), Helmholtz Zentrum München, Ingolstädter Landstrasse 1, Neuherberg 85764, Germany; 2 Chair for Biological Imaging, Technische Universität München, Trogerstr. 9, München 81675, Germany; 3 Department of Radiation Oncology, Klinikum rechts der Isar, Technische Universität München, Ismaninger Str. 22, München 81675, Germany; 4 CCG—Innate immunity in Tumor Biology, Helmholtz Zentrum München, Ingolstädter Landstrasse 1, Neuherberg 85764, Germany

## Abstract

Light propagating in tissue attains a spectrum that varies with location due to wavelength-dependent fluence attenuation, an effect that causes spectral corruption. Spectral corruption has limited the quantification accuracy of optical and optoacoustic spectroscopic methods, and impeded the goal of imaging blood oxygen saturation (sO_2_) deep in tissues; a critical goal for the assessment of oxygenation in physiological processes and disease. Here we describe light fluence in the spectral domain and introduce eigenspectra multispectral optoacoustic tomography (eMSOT) to account for wavelength-dependent light attenuation, and estimate blood sO_2_ within deep tissue. We validate eMSOT in simulations, phantoms and animal measurements and spatially resolve sO_2_ in muscle and tumours, validating our measurements with histology data. eMSOT shows substantial sO_2_ accuracy enhancement over previous optoacoustic methods, potentially serving as a valuable tool for imaging tissue pathophysiology.

## Abstract (web summary)

Spectral corruption impedes imaging of blood oxygen saturation. Here Tzoumas *et al.* describe light fluence in the spectral domain and introduce eigenspectra Multispectral Optoacoustic Tomography to account for wavelength-dependent light attenuation and estimate blood oxygen saturation within deep tissue.

## Graphical abstract

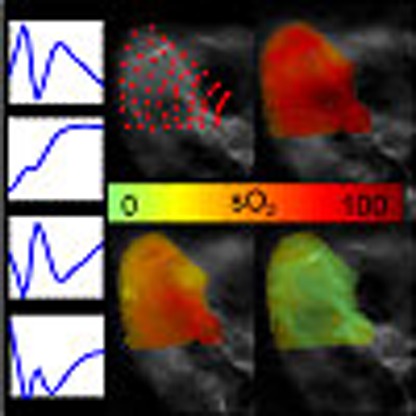

The assessment of tissue oxygenation is crucial for understanding tissue physiology and characterizing a multitude of conditions, including cardiovascular disease, diabetes, cancer hypoxia^1^ or metabolism. Today, measurements of the partial pressure of oxygen in tissue (pO_2_) and hypoxia measurements remain challenging and often rely on invasive methods that may change the tissue physiology, such as single-point needle polarography or immunohistochemistry^2^. Non-invasive imaging methods have been also considered, underscoring the importance of assessing pO_2_, but come with limitations. Positron emission tomography or single-photon emission computed tomography assess cell hypoxia through the administration of radioactive tracers^2^, but are often not well suited for quantifying pO_2_, suffer from low-spatial resolution and are unable to provide longitudinal or dynamic-imaging capabilities. Electron paramagnetic resonance imaging^3^ can measure tissue pO_2,_ but is not widely used and offers limited spatial and temporal resolution. Imaging methods using tracers may be further limited by restricted tracer bio-distribution, in particular to hypoxic areas. Tracer-free modalities have also been researched, in particular blood-oxygen-level dependent MRI^4^, which however primarily assesses only deoxygenated haemoglobin and, therefore, presents challenges in quantifying oxygenation and blood volume^5^.

Measurement of blood oxygen saturation levels (sO_2_) is a vital tissue physiology measurement and can provide an alternative way to infer pO_2_ and hypoxia. Arterial sO_2_ is widely assessed by the pulse oximeter, but this technology cannot be applied to measurements other than arterial blood. Optical microscopy methods like phosphorescence quenching microscopy^6^ or optoacoustic (photoacoustic) microscopy^7^ can visualize oxygenation in blood vessels and capillaries but are restricted to superficial (<1 mm depth) measurements. Diffuse optical methods received significant attention in the last two decades for sensing and imaging oxy- and deoxygenated haemoglobin deeper in tissue^8^. Despite recent progress^9^, diffuse optical methods are inherently limited in spatial resolution and accuracy, due to photon scattering. Owing to the high heterogeneity of blood sO_2_ in tissue, the values reported by diffuse optical methods are often hard to interpret.

Multispectral optoacoustic tomography (MSOT) detects the spectra of oxygenated and deoxygenated haemoglobin in high resolution deep within tissues, since signal detection and image reconstruction are not significantly affected by photon scattering^10^,^11^. Despite the principal MSOT suitability for non-invasive imaging of blood oxygenation, accuracy remains limited by the dependence of light fluence on depth and light colour. Unless explicitly accounted for, the wavelength-dependent light fluence attenuation with depth alters the spectral features detected and results in inaccurate estimates of blood sO_2_^12^,^13^. Despite at least two decades of research in optical imaging, the problem of light fluence correction has not been conclusively solved. To date, this problem has been primarily studied from an optical property quantification point of view^13^,^14^. However, it is not possible today to accurately image tissue optical properties *in vivo*, in high resolution, and compute light fluence^13^. Therefore, quantitative sO_2_ measurement deep in tissue *in vivo* remains an unmet challenge. Conventional spectral optoacoustic methods^15^,^16^ typically ignore the effects of light fluence and employ linear spectral fitting with the spectra of oxy- and deoxy-haemoglobin for estimating sO_2_ (linear unmixing), a common simplification that can introduce substantial errors in deep tissue.

In this work, we found that the spectral patterns of light fluence expected within the tissue can be modelled as an affine function of a few reference base spectra, independently of the specific distribution of tissue optical properties or the depth of the observation. We show how this principle can be employed to solve the spectral corruption problem without knowledge of the tissue optical properties, and significantly increase the accuracy of spectral optoacoustic methods. The proposed method, termed eigenspectra Multispectral Optoacoustic Tomography (eMSOT), can provide quantitative estimation of blood sO_2_ in deep tissue. We demonstrate the superior performance of the method with >2,000 simulations, phantom measurements and *in vivo* controlled experiments. Then, using eMSOT, we image oxygen gradients in the skeletal muscle *in vivo*, previously only accessible through invasive methods. Furthermore, we show the application of eMSOT in quantifying blood oxygenation gradients in tumours during tumour growth or O_2_ challenge, and relate label-free non-invasive eMSOT readings to tumour hypoxia; demonstrating the ability to measure quantitatively the perfusion hypoxia level in tumours, as confirmed with invasive histological gold standards.

## Results

### eMSOT concept and application

**Figure 1:**
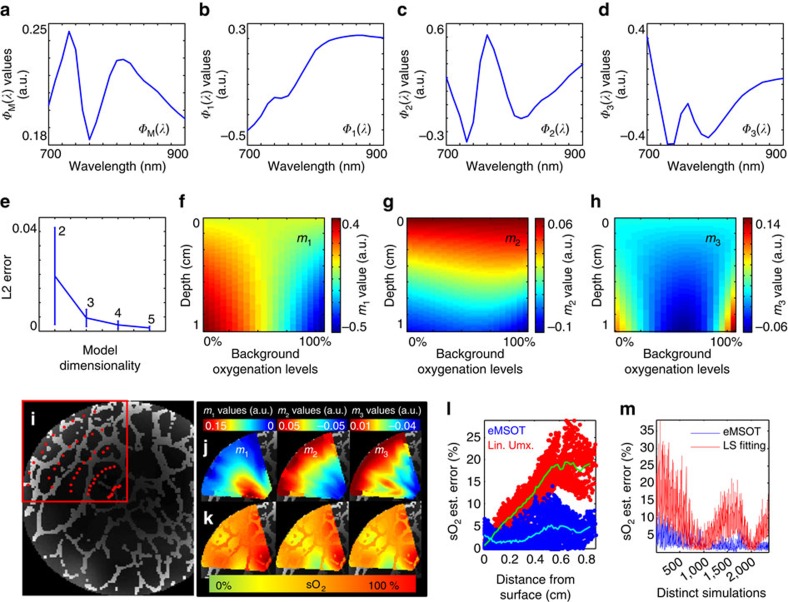
eMSOT concept and application. (**a**–**d**) The Eigenspectra model composed of a mean fluence spectrum *Φ*_M_(*λ*) (**a**) and the three fluence Eigenspectra *Φ*_1_(*λ*), *Φ*_2_(*λ*) and *Φ*_3_(*λ*), (**b**–**d**), respectively, as derived by applying PCA on a selected training data set of light fluence spectra (Methods). (**e**) Statistics of the L2 norm error of the Eigenspectra model on the training data set for different model dimensionalities. Error bar denotes s.d. (**f**–**h**) Values of the parameters *m*_1_, *m*_2_ and *m*_3_ as a function of tissue depth (*y* axis) and sO_2_ (*x* axis). The values have been obtained after fitting the light fluence spectra of the training data set (see Methods) to the Eigenspectra model. (**i**) Application of a circular grid (red points) for eMSOT inversion on an area of a simulated MSOT image. (**j**) After eMSOT inversion the model parameters *m*_1_, *m*_2_ and *m*_3_ are estimated for all grid points and maps of *m*_1_, *m*_2_ and *m*_3_ are produced for the convex hull of the grid by means of cubic interpolation. These maps are used to spectrally correct the original MSOT image. (**k**) Blood sO_2_ estimation using linear unmixing (left), eMSOT (middle) and Gold standard sO_2_ (right) of the selected region. (**l**) sO_2_ estimation error of the analysed area sorted per depth in the case of linear unmixing (red points) and eMSOT (blue points). (**m**) Mean sO_2_ error of linear unmixing (red) and eMSOT (blue) corresponding to >2,000 simulations of random structures and optical properties (see Supplementary Note 3).

A new concept of treating light fluence in diffusive media/tissues is introduced, based on the observation that the light fluence spectrum at different locations in tissue is the result of a cumulative light absorption operation by tissue chromophores, such as haemoglobin. We, therefore, hypothesized that there exists a small number of base spectra that can be combined to predict any fluence spectrum present in tissue; therefore, avoiding the unattainable task of knowing the distribution of tissue optical properties at high resolution. To prove this hypothesis, we first applied principal component analysis (PCA) on 1,470 light fluence spectral patterns, which were computed by simulating light propagation in tissues at 21 different (uniform) oxygenation states of haemoglobin and 70 different discrete depths (Methods). PCA analysis yielded four significant base spectra, that is, a mean light fluence spectrum (Fig. 1a) and three fluence Eigenspectra (Fig. 1b–d). PCA was used due to its optimality in modelling the spectral variability of light fluence in a linear manner (see Methods). We found that the selection of three Eigenspectra offers a simple model with relatively high-modelling accuracy (Fig. 1e).

We then postulated that light fluence spectra in arbitrary and non-uniform tissues can be modelled as a superposition of the mean fluence spectrum (*Φ*_M_) and the three Eigenspectra (*Φ*_*i*_(*λ*), *i*=1..3) multiplied by appropriate scalars *m*_1_, *m*_2_ and *m*_3_, termed Eigenfluence parameters. To validate this hypothesis we computed the light fluence in >500 simulated tissue structures of different and non-uniform optical properties and haemoglobin oxygenation values (Supplementary Note 1). For each pixel, we fitted the simulated light fluence spectrum to the Eigenspectra model and derived the Eigenfluence parameters (*m*_1_, *m*_2_, *m*_3_) and a fitting residual value. The residual value represents the error of the Eigenspectra model in matching the simulated data and typically assumed values below 1% (see Supplementary Note 1, Supplementary Fig. 1), indicating that three Eigenspectra can accurately model all simulated fluence spectra generated in tissues of arbitrary structure. We further observed that the values of *m*_2_ vary primarily with tissue depth while the values of *m*_1_, *m*_3_ also depend on the average levels of background blood sO_2_ (see Fig. 1f–h). Intuitively, this indicates that the second Eigenspectrum *Φ*_2_(*λ*) is mainly associated with the modifications of the light fluence spectrum due to depth and the average optical properties of the surrounding tissue, while the first Eigenspectrum *Φ*_*1*_(λ) is also associated with the ‘spectral shape' of light fluence that relates to the average oxygenation of the surrounding tissue. Localized measurements of light fluence spectra obtained *in vivo* and *post mortem* corroborated these observations (Supplementary Fig. 2).

Following these observations, we propose eigenspectra MSOT (eMSOT), based on three eigenspectra *Φ*_1_(*λ*), *Φ*_2_(*λ*), *Φ*_3_(*λ*), as a method that formulates the blood sO_2_ estimation problem as a nonlinear spectral unmixing problem (see Methods), that is:


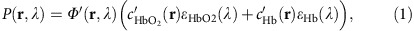


where *P*(**r**,*λ*) is the multispectral optoacoustic image intensity obtained at a position **r** and wavelength *λ*, 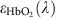 and *ɛ*_Hb_(*λ*) are the wavelength-dependent molar extinction coefficients of oxygenated and deoxygenated haemoglobin, 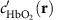 and *c′*_Hb_(**r**) are the relative concentrations of oxygenated and deoxygenated haemoglobin (proportional to the actual ones with regard to a common scaling factor, see Methods), and *Φ′*(**r,***λ*)=*Φ*_M_(*λ*)+*m*_1_(**r**)*Φ*_1_(*λ*)+*m*_2_(**r**)*Φ*_2_(*λ*)+*m*_3_(**r**)*Φ*_3_(*λ*). Equation (1) defines a nonlinear inversion problem, requiring measurements at 5 wavelengths or more for recovering the five unknowns, that is, 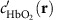, *c′*_Hb_(**r**), *m*_1_(**r**), *m*_2_(**r**), *m*_3_(**r**) and is solved as a constrained optimization problem (see Methods, Supplementary Note 2, Supplementary Fig. 3). Since the light fluence varies smoothly in tissue, we only compute the Eigenfluence parameters on a coarse grid subsampling the region of interest (Fig. 1i), for computational efficiency. Then, cubic interpolation is employed to compute the Eigenfluence parameters in each pixel within the convex hull of the grid (Fig. 1j) and calculate a fluence spectrum *Φ′*(**r,***λ*) for each pixel using these parameters. Equation (1) is then solved for 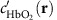 and *c′*_Hb_(**r**), and sO_2_ is computed (see Methods).

The performance of eMSOT was validated using simulated data obtained from a light propagation model (finite element solution of the diffusion equation) applied on >2,000 randomly created maps of different optical properties, simulating different tissue physiological states (Supplementary Note 3). Figure 1k depicts a representative example of a simulated blood sO_2_ map and visually showcases the differences between the eMSOT sO_2_ image (middle), the sO_2_ image obtained using linear unmixing (left), and the original sO_2_ simulated image (right). eMSOT offered significantly lower sO_2_ estimation error with depth, compared with the linear fitting method (Fig. 1l). A substantially improved sO_2_ estimation accuracy was observed using eMSOT over linear unmixing when we analysed the complete simulation data set (Fig. 1m). In particular, for imaging tissue depths of >5 mm eMSOT offered a 3–8-fold sO_2_ estimation improvement over linear unmixing (Supplementary Fig. 4n). A thorough validation of eMSOT performance across different data sets, optical properties and grid densities is presented in Supplementary Note 3, Supplementary Tables 1 and 2, and Supplementary Fig. 4.

**Figure 2:**
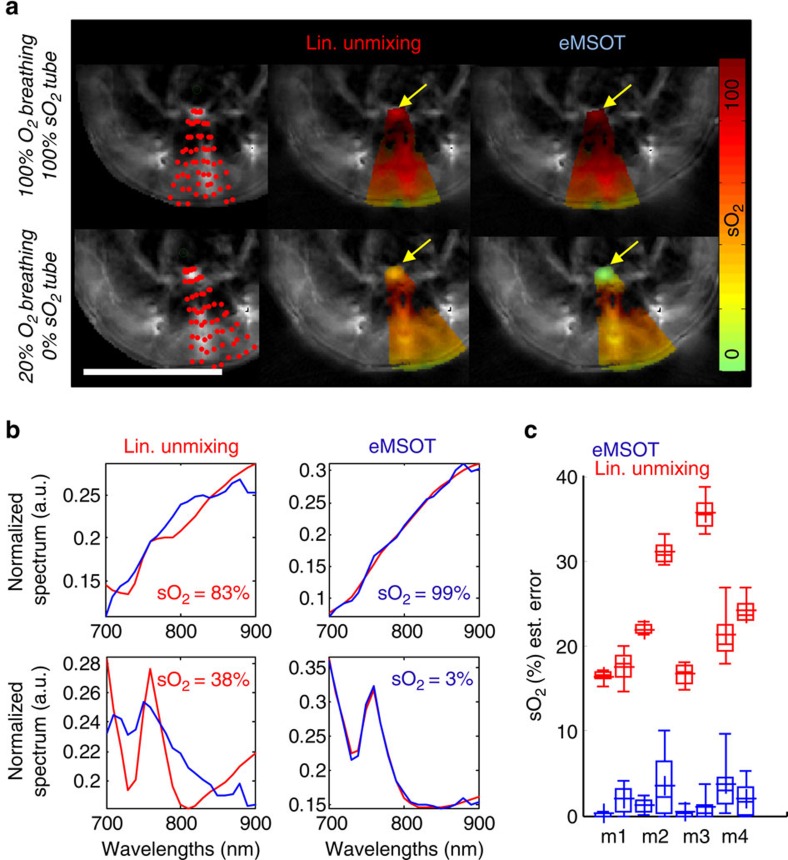
Comparison of eMSOT sO_2_ estimation accuracy over conventional spectral optoacoustic method. (**a**) eMSOT application in the case of *in vivo* controlled experiments under 100% O_2_ (**a** upper row) and 20% O_2_ (**a** lower row) breathing. Capillary tubes containing blood of 100% sO_2_ (upper row) and 0% sO_2_ (lower row) were inserted within tissue (arrows). Scale bar, 1 cm. (**b**) Spectral fitting and sO_2_ estimation in the insertion area (yellow arrows in **a**) using linear unmixing (left column) and eMSOT (right column). The blue curves correspond to *P*(**r**,*λ*) (left column) and *P*^eMSOT^(**r**,*λ*) (right column) while the red curves correspond to 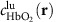

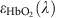
+*c*_Hb_^lu^(**r**)ɛ_Hb_(*λ*) (left column; the term lu refers to linear unmixing) and 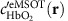

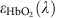
+*c'*_Hb_^eMSOT^(**r**)*ɛ*_Hb_(*λ*) (right column). (**c**) sO_2_ estimation error using eMSOT (blue) and linear unmixing (red) in all four animal experiment repetitions m1–m4. Two values are reported for each experiment corresponding to a 100% sO_2_ insertion (left) and 0% sO_2_ insertion (right). Statistics are derived from all pixels in a region of interest (ROI) corresponding to the insertion area for each data set. The boxes include 25–75% and the error bars 9–91% of the data. The mean value is denoted with the plus symbol.

For experimentally assessing the accuracy of eMSOT, we performed a series of blood phantom experiments that suggest an up to 10-fold more reliable sO_2_ estimation derived by eMSOT, as compared with conventional linear unmixing (Supplementary Note 4, Supplementary Fig. 5). In addition, controlled mouse measurements (*n*=4) were performed *in vivo,* under gas anaesthesia, by rectally inserting capillary tubes containing blood at 100 and 0% sO_2_ levels (Methods). The mice were imaged in the lower abdominal area under 100% O_2_ and 20% O_2_ breathing conditions (Fig. 2a). Figure 2a presents the eMSOT grid applied on the images processed (left column), the sO_2_ maps obtained with linear unmixing (middle column) and with eMSOT (right column). The spectral fitting of linear unmixing (left) and eMSOT (right) corresponding to a pixel in the area of the capillary tube (yellow arrows in a) are presented in Fig. 2b along with the estimated sO_2_ values. In the controlled *in vivo* experiments, the mean linear unmixing error ranged from 16 to 35% while eMSOT offered a mean sO_2_ error ranging from 1 to 4%, indicating an order of magnitude improved accuracy (Fig. 2c).

### Imaging blood oxygenation gradients in muscle and tumour

Blood oxygenation and oxygen exchange in the microcirculation have been traditionally studied through invasive, single-point polarography or microscopy measurements in vessels and capillaries of the skeletal muscle^17^. Research for macroscopic methods that could non-invasively resolve muscle oxygenation was broadly pursued in the past two decades by considering near-infrared spectroscopy and diffuse optical tomography, which, however can only report bulk tissue sO_2_ values^18^,^19^. In a next set of experiments we, therefore, studied whether eMSOT could non-invasively quantify the oxygenation gradient in the skeletal muscle, and we compared this performance to conventional spectral optoacoustic methods. eMSOT was applied in the area of the hindlimb muscle of mice undergoing an O_2_ challenge as described in Supplementary Note 5 (*n*=6 animal experiments); three of the mice were then killed with an overdose of CO_2_, the latter binding to haemoglobin and deoxygenating blood.

**Figure 3:**
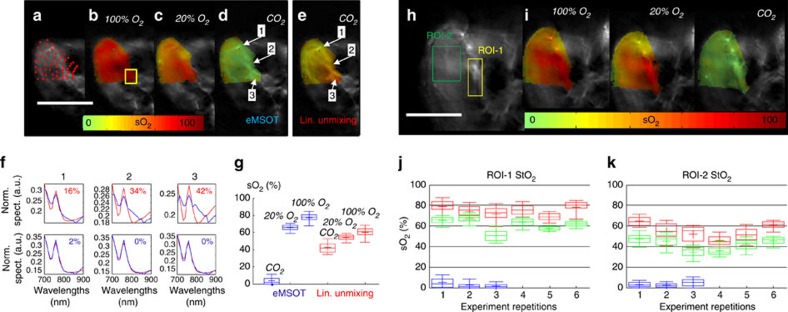
eMSOT measurements of tissue blood oxygenation in the muscle. (**a**–**d**) eMSOT grid applied on the area of the hindlimb muscle (**a**) and eMSOT tissue blood sO_2_ estimation in the case of 100% O_2_ breathing (**b**), 20% O_2_ breathing (**c**) and *post mortem* after CO_2_ breathing (**d**). (**e**) sO_2_ estimation using linear unmixing in the *post mortem* case after CO_2_ breathing. Scale bar, 1 cm. (**f**) Normalized spectra, spectral fitting and sO_2_ values of linear unmixing (upper row) and eMSOT (lower row) for the three points indicated in (**d**,**e**). The blue curves correspond to *P*(**r**,*λ*) (upper row) and *P*^eMSOT^(**r**,*λ*) (lower row) while the red curves correspond to 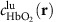

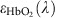
+*c*_Hb_^lu^(**r**)*ɛ*_Hb_(*λ*) (upper row) and 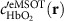

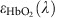
+*c'*_Hb_^eMSOT^(**r**)*ɛ*_Hb_(*λ*) (lower row). (**g**) Estimated blood sO_2_ of a deep tissue area (yellow box in (**b**)) using eMSOT (blue) and linear unmixing (red) under different breathing conditions of CO_2_, 20% O_2_ and 100% O_2_. (**h**) Anatomical MSOT image of the hindlimb area at an excitation wavelength of 900 nm. Two regions were selected for presenting the sO_2_ values, one close to prominent vasculature (ROI-1) and one corresponding to soft tissue (ROI-2). Scale bar, 0.5 cm. (**i**) eMSOT sO_2_ estimation *in vivo* under 100% (left) and 20% O_2_ breathing (middle) and *post mortem* after CO_2_ breathing (right). Scale bar, 0.5 cm. (**j**,**k**) Estimated tissue sO_2_ of ROI-1 (**j**) and ROI-2 (**k**) under 100% (red) and 20% O_2_ breathing (green) and *post mortem* after CO_2_ breathing (blue). The *x* axis positions correspond to six different animal experiment repetitions. Statistics in (**g**,**j**,**k**) are derived from all pixels in the corresponding ROIs. The boxes include 25–75% and the error bars 9–91% of the data. The mean value is denoted with the plus symbol.

eMSOT applied in the hindlimb muscle area (grid shown in Fig. 3a) resolved oxygenation gradients as a function of breathing conditions *in vivo* (Fig. 3 b,c) and *post mortem* after CO_2_ breathing (Fig. 3d). The *post-mortem* deoxygenated muscle served herein as a control experiment and was also analysed with linear unmixing for comparison (Fig. 3e). In the *post-mortem* case, linear unmixing overestimated the sO_2_ as a function of tissue depth (Fig. 3e) and yielded large errors in matching the tissue spectra (Fig. 3f—upper row). Conversely, eMSOT offered sO_2_ measurements in agreement with the expected physiological states (Fig. 3b–d) and consistently low-fitting residuals (Fig. 3f—lower row, Supplementary Fig. 6). Figure 3d–f demonstrates the prominent effects of spectral corruption with depth that impair the accuracy of conventional spectral optoacoustic methods, but are tackled by eMSOT. The estimated blood sO_2_ values corresponding to a deep tissue area (yellow rectangle in Fig. 3b) are tabulated in Fig. 3g for eMSOT and linear unmixing, and depict that the latter demonstrated unrealistically small sO_2_ changes between the normoxic *in vivo* and anoxic *post-mortem* (after CO_2_ breathing) states.

In addition to physiological tissue features, MSOT also reveals tissue morphology. MSOT images at a single wavelength (900 nm) captured prominent vascular structures likely corresponding to femoral vessels or their branches (Fig. 3h) with implicitly co-registered eMSOT blood oxygenation images (Fig. 3i). This hybrid mode enables the study of physiology at specific tissue areas. We selected to study blood oxygenation measurements at a region of interest around large vessels (ROI-1; Fig. 3h) and a region of interest within the muscle presenting no prominent vascular structures (ROI-2; Fig. 3h) for the 100% O_2_, 20% O_2_ and CO_2_ breathing conditions. Average tissue sO_2_ was typically measured at 60–70% saturation under medical air breathing and at 70–80% saturation under 100% O_2_ breathing near large vessels (Fig. 3j). Average tissue blood oxygenation away from large vessels (ROI-2) was estimated consistently lower, at 35–50% saturation under normal breathing conditions, and 45–60% saturation under 100% O_2_ breathing (Fig. 3k).

The low blood saturation values in tissue (35–50%) cannot be explained by considering arterial and venous blood saturation. However, previous studies based on direct microscopy measurements in vessels and capillaries through polarography, haemoglobin spectrophotometry and phosphorescence quenching microscopy have revealed similar oxygenation gradient in the skeletal muscle^17^ with haemoglobin saturation in the femoral artery found to range between 87 and 99% sO_2_^17^,^20^, while rapidly dropping down to 50–60% sO_2_ in smaller arterioles^20^,^21^. The average oxygen saturation in venules and veins has been found to range between 45% and 60% sO_2_ under normal breathing conditions, reaching up to 70% at 100% O_2_ breathing^21^,^22^. Average capillary blood oxygenation has been estimated at 40% sO_2_ with a large standard deviation^22^, often reported lower, at an average, than venular oxygenation^17^. Therefore, the eMSOT values measured at ROI-1 possibly relate to a weighted average of arterial/arteriolar and venous/venular sO_2_ in the skeletal muscle, while the values measured at ROI-2, which anatomically presents no prominent vasculature, relate more to capillary sO_2_ measurements.

**Figure 4:**
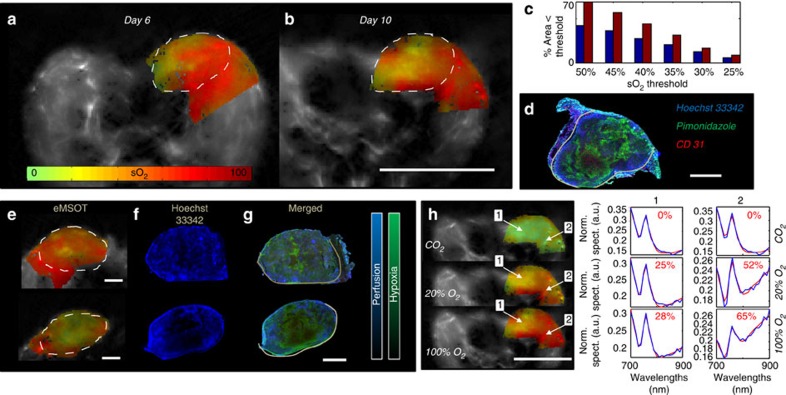
eMSOT measurements of tissue blood oxygenation in tumour. (**a**,**b**) sO_2_ maps of a 4T1 tumour implanted in the mammary pad at day 6 (**a**) and day 10 (**b**) after cell inoculation. Dashed lines represent a segmentation of the tumour area. Scale bar, 1 cm. (**c**) Bar-plot presenting the percentage of the total tumour area containing sO_2_ values lower than a specific sO_2_ threshold (*x* axis). Blue bars correspond to the tumour imaged at day 6 and red bars correspond to the tumour imaged at day 10, presented in (**a**,**b**). (**d**) Merged Hoechst 33342, CD 31 and Pimonidazole staining of the tumour presented in (**b**). Scale bar, 2 mm. (**e**,**g**) Examples of a highly perfused (upper row) and a low perfused (lower row) tumour analysed with eMSOT for sO_2_ estimation (**e**) Hoechst 33342 staining (**f**) and merged with Pimonidazole staining (**g**). Tumour areas presenting lower sO_2_ values in eMSOT measurements also showed lower Hoechst 33342 signal intensity, representing reduced perfusion in these areas. Scale bar, 2 mm. ((**h**) left) sO_2_ maps of a tumour under an O_2_–CO_2_ challenge. Scale bar, 1 cm. The computed sO_2_ values and the eMSOT spectral fit of points 1 and 2 (arrows) are presented in ((**h**) right) for the three breathing conditions. The blue curves correspond to *P*^eMSOT^(**r**,*λ*) while the red curves correspond to 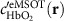

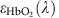
+*c'*_Hb_^eMSOT^(**r**)*ɛ*_Hb_(*λ*).

The improved accuracy observed in eMSOT over previous approaches and general agreement with invasive tissue measurements prompted the further study of perfusion hypoxia emerging from the incomplete delivery of oxygenated haemoglobin in tissue areas. We hypothesized that measurements of blood saturation could be employed as a measure of tissue hypoxia, assuming natural haemoglobin presence in hypoxic areas. To examine this hypothesis, we applied eMSOT to measure blood oxygenation in 4T1 solid tumours orthotopically implanted in the mammary pad of eight mice (Methods, Supplementary Note 6). MSOT revealed the tumour anatomy and eMSOT exposed tumour heterogeneity, which was found consistent to anatomical features identified through cryoslice colour photography and haematoxil and eosin staining (Supplementary Note 6, Supplementary Fig. 7c–g). Furthermore, imaging tumours at different time-points revealed the progression of hypoxia during tumour growth (Fig. 4a,b). The spread of hypoxia, that is, the percentage of the hypoxic area (area presenting sO_2_ values below a threshold which varied from 50 to 25% sO_2_) over the total tumour area also increased during tumour progression (Fig. 4c). Following the *in vivo* measurements we harvested the tumour tissue and related the non-invasive eMSOT findings to the histological assessment of tumour hypoxia (see Supplementary Note 6 and Supplementary Fig. 7). Tumour tissue was stained by Hoechst 33342 (ref. 23) (indicating perfusion) and Pimonidazole^24^ (indicating cell hypoxia). The results indicated close correspondence between the hypoxic areas detected by eMSOT using haemoglobin as a hypoxia sensor (Fig. 4b) and the histology slices (Fig. 4d). We found that eMSOT could not only quantitatively distinguish between high- and low-hypoxia levels in the tumours, but the spatial sO_2_ maps further presented congruence with the spatial appearance of hypoxia spread and reduced perfusion seen in the histology slices (Fig. 4e–g). A quantitative congruence analysis is shown in Supplementary Figure 7. Finally, clear differences were also observed between the hypoxic tumour and healthy tissue response to an O_2_ breathing challenge (Fig. 4h; Supplementary Fig. 8), with areas in the core of the tumour presenting a limited response to such external stimuli, likely due to the presence of non-functional vasculature.

## Discussion

Spectral corruption has so far limited the potential of optical and optoacoustic methods to offer accurate, quantitative assessment of sO_2_ deep inside tissues. Conventional computational methods in optical imaging propose to invert a light transport operator to recover tissue optical properties (absorption and scattering)^13^; then use these properties for calculating tissue physiological parameters. However, the very-high numerical complexity and ill-posed nature of the inversion problem has not allowed so far accurate, high-resolution sO_2_ imaging. We hereby followed an alternative approach that describes the spectral features of light fluence as a combination of spectral base functions. Using this principle, we formulated the sO_2_ quantification problem as a nonlinear spectral unmixing problem that does not require knowledge of tissue optical properties or the inversion of a light transport operator. Effectively, eMSOT converts sO_2_ imaging from a problem that is spatially dependent on light propagation and optical properties, as common in traditional optical methods, to a problem solved in the spectral domain. Therefore, sO_2_ can be directly quantified without estimating tissue optical properties.

eMSOT requires theoretically at least five excitation wavelengths for resolving spectral domain parameters and the relative oxygenated and deoxygenated haemoglobin concentrations. We hereby utilized 21 wavelengths for ensuring high accuracy. The recent evolution of video-rate MSOT imaging systems, based on fast tuning optical parametric oscillator lasers^25^, allows the practical implementation of the method. Modern MSOT systems offer five wavelength scans at 20 Hz or better. Therefore eMSOT is a technology that optimally interfaces to a new generation of fast and handheld spectral optoacoustic systems^26^.

The method developed demonstrated quantitative, non-invasive blood oxygenation images in phantoms and tissues *in vivo* (muscle and tumour) in high resolution, showing good correlation with the expected physiological state or the histologically observed spatial distribution of perfusion and hypoxia. eMSOT measures blood oxygenation. We hypothesized that a correlation exists to tissue oxygenation and hypoxia measurements by assuming a wide presence of haemoglobin in tissues. We demonstrated congruence (Supplementary Note 6) between traditional invasive histological assays resolving tissue hypoxia and eMSOT analysis. Importantly, not only average values are resolved, but there is a close spatial correspondence between hypoxia patterns resolved by eMSOT non-invasively and histological analysis (Fig. 4, Supplementary Fig. 7).

High-resolution non-invasive imaging of blood oxygenation across entire tissues and tumours offers novel abilities in studying physiological and pathological conditions. This goal has been pursued for decades with near-infrared methods, but the strong effects of photon scattering and photon diffusion on the signals detected limited imaging resolution and often impeded accurate quantification^27^. Optoacoustic imaging improves the resolution achieved, over diffuse optical imaging methods but its sO_2_ estimation accuracy has been limited so far by depth-dependent fluence attenuation and spectral corruption effects. We showed that conventional spectral optoacoustic methods employing linear unmixing can significantly misestimate blood oxygen saturation values in several cases, including simulations and controlled animal measurements. eMSOT was tested on a vast data set consisting of >2,000 tissue simulations and was consistently found to provide from a comparable to substantially better sO_2_ estimation accuracy over linear unmixing. (Supplementary Note 3). The large number of simulations was necessary to validate eMSOT, which presents a non-convex optimization problem. eMSOT was further tested on tissue mimicking blood phantoms (Supplementary Note 4) and controlled *in vivo* experiments (Fig. 2, Supplementary Note 5). In all cases tested, eMSOT offered from comparable to significantly more accurate performance over conventional spectral optoacoustic methods.

A particular challenge in this study was the confirmation of the eMSOT values obtained *in vivo*. Polarography measurements are invasive, disrupt the local microenvironment and do not allow to recover spatial information. Nuclear methods using tracers are not well suited for longitudinal studies and utilize tracers that need to distribute in hypoxia areas, that is, areas with problematic supply. Therefore, the results may not directly compare to eMSOT, even though such study is planned as a next step. Blood-oxygen-level dependent MRI only resolves the effects of deoxygenated haemoglobin but cannot observe oxygenated haemoglobin. For this reason, we selected to utilize traditional histology methods, using cryoslicing, which allows to maintain spatial orientation so that eMSOT and histological results could be compared not only in terms of quantity but also in regard to the spatial appearance.

eMSOT proposes a solution to a fundamental challenge in optical and optoacoustic imaging. In the absence of established and reliable methods that can image blood oxygenation, it may be that eMSOT becomes the gold standard method in blood sO_2_ studies. Its congruence with tissue hypoxia may also allow a broad application in tissue and cancer hypoxia studies. Nevertheless eMSOT performs optimally when applied on well-reconstructed parts of optoacoustic images (Supplementary Note 5). For this reason, it was selectively applied herein to the part of the image that is within the optimal sensitivity field of the detector employed. An eMSOT advantage is that it is insensitive to scaling factors such as the Grüneisen coefficient or the spatial sensitivity field of the imaging system (Methods). However, due to its scale invariance eMSOT only allows for quantifying blood sO_2_ and not absolute blood volume, a goal that will be interrogated in future studies. Next steps further include the eMSOT validation with a larger pool of tissue physiology interrogations spanning from cancer, cardiovascular and diabetes research, relation of physiological phenotypes to metabolic and ‘-omic' outputs and in clinical application.

## Methods

### Animal preparation and handling

All procedures involving animal experiments were approved by the Government of Upper Bavaria. For the preparation of orthotopic 4T1 tumour models, 8-week-old, adult female, athymic, Nude-Foxn1 mice (Harlan, Germany) were orthotopically inoculated in the mammary pad with cell suspensions (0.5 million 4T1 cells (CRL-2539)). Animals (*n*=8) were imaged *in vivo* using MSOT after the tumours reached a suitable size. All imaging procedures were performed under anaesthesia using 1.8% isoflurane. In the O_2_ challenge experiment, the mouse was initially breathing 100% O_2_ and in the following medical air (20% O_2_). During the O_2_ Challenge, the mice were stabilized for a period of 10 min under each breathing condition before MSOT acquisition. For controlled mouse measurements (*n*=4), MSOT acquisition was performed on mice under gas anaesthesia and breathing 100% O_2_ or 20% O_2_ by rectally inserting a capillary tube containing pig blood at 100 or 0% sO_2_ oxygenation levels. Mice were killed during MSOT imaging with an overdose of CO_2_ or after MSOT acquisition by a ketamine/xylazine overdose. In the following the mice were stored at −80 °C for further analysis.

4T1 cell line was acquired from American type culture collection (ATCC-CRL-2539, #5068892). The cells were authenticated by the Americal type culture collection (ATCC) by several analysis tests: post-freeze viability, morphology, mycoplasma contamination, post-freeze cell growth, interspecies determination; bacteria and fungal contamination. Additional mycoplasma contamination tests were also performed. For the animal studies no randomization, blinding or statistical methods were performed.

### MSOT imaging

Optoacoustic imaging was performed using a real-time whole-body mouse imaging scanner, MSOT In Vision 256-TF (iThera-Medical GmbH, Munich, Germany). The system utilizes a cylindrically focused 256-element transducer array at 5 MHz central frequency covering an angle of 270 degrees. The system acquires cross-sectional (transverse) images through the animal. The animals are placed onto a thin-clear polyethylene membrane. The membrane separates the animals from a water bath, which is maintained at 34 °C and is used for acoustic coupling and maintaining animal temperature while imaging. Image acquisition speed is at 10 Hz^28^. Imaging was performed at 21 wavelengths from 700 to 900 nm with a step size of 10 nm, and at 20 consecutive slices with a step size of 0.5 mm. Image reconstruction was performed using a model-based inversion algorithm^29^,^30^ with a non-negativity constraint imposed during inversion and with Tikhonov regularization.

### eMSOT method and sO_2_ maps

All optoacoustic images *P*(**r**,*λ*) obtained over excitation wavelength *λ* were calibrated to correct for the intensity of laser power per pulse, and for the absorption of water surrounding the tissue. With HbO_2_ and Hb being the main tissue absorbers in the near-infrared, multispectral optoacoustic images can be related to the concentrations of oxy- and deoxy-haemoglobin through equation (2).


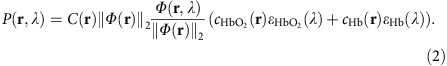


In equation (2), *Φ*(**r**,λ) is the space and wavelength-dependent optical fluence, *C*(**r**) is a spatially varying scaling factor corresponding to the effects of the system's spatial sensitivity field and the Grüneisen coefficient, 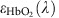 and *ɛ*_Hb_(*λ*) are the wavelength-dependent molar extinction coefficients of oxygenated and deoxygenated haemoglobin, while 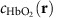 and *c*_Hb_(**r**) the associated concentrations at a position **r**. **Φ(r)** is a vector corresponding to the light fluence spectrum at position **r**, and ||**Φ**(**r**)||_2_ is its norm across all excitation wavelengths at a position **r**. We define *Φ*′(**r,***λ*)=*Φ*(**r,***λ*)/||**Φ**(**r**)||_2,_ which corresponds to the normalized wavelength dependence of light fluence at a specific position (that is, normalized spectrum of light fluence).

The space-only dependent factors *C*(**r**) and ||**Φ**(**r**)||_2_ do not affect the estimation of blood sO_2,_ which is calculated as a ratio once the relative concentrations of HbO_2_ and Hb are known. We define 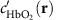
=*C′*(**r**)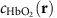 and *c′*_Hb_(**r**)=*C′*(**r**)c_Hb_(**r**), respectively, where *C′*(**r**)=*C*(**r**)||**Φ**(**r**)||_2_ is a common, space-only dependent scaling factor. Using this notation, equation (2) can be transformed into equation (1). Given 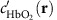 and *c′*_Hb_(**r**), sO_2_ can be computed as in:


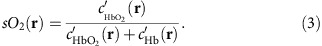


For the accurate quantitative extraction of the relative concentrations 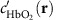 and *c′*_Hb_(**r**), accounting for, or estimating the wavelength dependence of the light fluence *Φ′*(**r,***λ*) is further required.

*The Eigenspectra model*. eMSOT is based on the observation that the spectral patterns of light fluence present in tissue can be modelled as an affine function of only a few base spectra, independently of tissue depth and the specific distribution of optical properties of the tissue imaged. This hypothesis stems from the notion that the spectrum of light fluence is the result of the cumulative light absorption by haemoglobin; thus the spectrum of light fluence will always be related to the spectra of haemoglobin in a complex nonlinear manner. This complex relation can be linearized using a data-driven approach, that is, through the application of PCA on a selected set of light fluence spectra.

The wavelength dependence of the light fluence was herein modelled as a superposition of a mean fluence spectrum *Φ*_Μ_(*λ*) and a linear combination of a number of light fluence Eigenspectra *Φ*_*i*_(*λ*). This model was derived by applying PCA on a training data set comprised of a set of light-fluence spectral patterns. Briefly, a training data set was formed through the creation of multispectral light fluence simulations using the 1D analytical solution of the diffusion equation for infinite media. A set of light-fluence spectral patterns *Φ*_*z,ox*_(*λ*) were computed for high physiological tissue optical properties (*μ*_*α*_=0.3 cm^−1^, *μ*_*s*_*′*=10 cm^−1^), tissue depths ranging from *z*=0 to *z*=1 cm with a step size of 0.143 mm (70 discrete depths in total) and for 21 different uniform background blood sO_2_ levels (*ox* ε {0%, 5%, 10, …, 100%}). The so computed set of light fluence spectra *Φ*_*z,ox*_(*λ*) was normalized (*Φ'*_*z,ox*_(*λ*)=*Φ*_*z,ox*_(*λ*)/||**Φ**_**z,ox**_||_2_) and used in the following as training data in the context of PCA in order to create an affine, 3-dimensional model consisting of a mean fluence spectrum *Φ*_Μ_(*λ*) and three Eigenspectra *Φ*_i_(*λ*). PCA was used for offering a minimum square error property in capturing the spectral variability of the simulated light fluence spectra, in a linear manner. Three components were selected for providing a relatively simple model while also offering a small model error with respect to the training data set (Fig. 1e). The wavelength dependence of the light fluence *Φ′*(**r,***λ*) at any arbitrary tissue position **r** can thus be modelled as a superposition of the mean fluence spectrum and three fluence Eigenspectra multiplied by appropriate scalar parameters *m*_1_(**r**), *m*_2_(**r**) and *m*_3_(**r**), (hereby referred to as Eigenfluence parameters) as per equation (4):


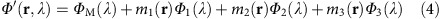


The so created 3-dimensional affine forward model of the wavelength dependence of light fluence was tested with regard to light-fluence spectral patterns produced in completely heterogeneous media with varying and randomly distributed optical properties and oxygenation values and demonstrated high accuracy (Supplementary Fig. 1). The forward model was further tested through *in vivo* and *ex vivo* light fluence measurements, obtained from controlled experiments (Supplementary Fig. 2).

Through simulations, it was observed that the values of the *m*_2_ Eigenfluence parameter relate primarily to tissue depth and the average tissue optical properties. This trend was observed both in the case of tissue simulations with uniform optical properties (Fig. 1g), as well as in complex and randomly created tissue simulations described in Supplementary Note 1, 3. Conversely, the values of the Eigenfluence parameters *m*_1_ and *m*_3_ relate both to tissue depth, as well as to tissue background oxygenation. Specifically both *m*_1_ and *m*_3_ present a trend of increasing absolute values with depth and a sign that relates to background blood sO_2_. These observations were confirmed with *in vivo* and *ex vivo* light fluence measurement experiments (Supplementary Note 1).

*Model inversion*. Using the Eigenspectra model of light fluence, the blood sO_2_ quantification problem at a position **r** formulates as the problem of estimating 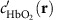 and *c′*_Hb_(**r**) by minimizing *f*(**r;** *m*_1_(**r**), *m*_2_(**r**), *m*_3_(**r**), 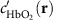, *c′*_Hb_(**r**)), for brevity noted *f*(**r**), defined according to equation (5):


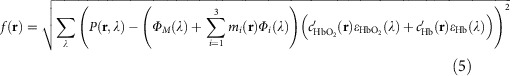


A solution for the five unknowns (namely the three light fluence model parameters, *m*_1…3_(**r**) and the relative blood concentrations 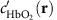 and *c′*_Hb_(**r**)) can be obtained using a nonlinear optimization algorithm and at least five excitation wavelengths. The relative blood concentrations 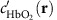 and *c′*_Hb_(**r**) are proportional to the actual ones (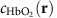 and *c*_Hb_(**r**)) with regard to a common scaling factor. However, as stated before, this fact does not affect the computation of sO_2_.

The minimization problem defined by equation (5) is ill-posed and may converge to a wrong solution unless properly constrained. For achieving inversion stability and accurate sO_2_ estimation results, the cost function *f* of equation (5) is simultaneously minimized in a set of grid points placed in the image domain (Fig. 1i), where three-independent constraints are further imposed to the Eigenfluence parameters. These constraints correspond to the relation of the Eigenfluence parameters between neighbour grid points and to the allowed search space for the Eigenfluence parameters:

- Since the values of the second Eigenfluence parameter *m*_2_ present a consistent trend of reduction with tissue depth observed both in the case of uniform tissue simulations (see Fig. 1g), as well as in simulations with random structures, *m*_2_ is constrained to obtain smaller values in the case of grid points placed deeper into tissue.
- As the light fluence spectrum is bound to vary smoothly in space due to the nature of diffuse light propagation, large variations of the Eigenfluence parameters *m*_1_, and *m*_3_ between neighbour pixels are penalized. This spatial smoothness constraint is achieved through the incorporation of appropriate regularization parameters *α*_*i*_ to the cost function for constraining the variation of the model parameters (see equation (6)). The values of the regularization parameters were selected using cross-validation on simulated data sets (Supplementary Note 2).
- Since the values of *m*_1_ and *m*_3_ are strongly dependent on background blood sO_2_, an initial less accurate estimation of tissue sO_2_ can be effectively used to reduce the total search space to a constrained relevant sub-space. The limits of search space for the Eigenfluence parameters *m*_1_ and *m*_3_ corresponding to each grid point are identified in a preprocessing step as analytically described in Supplementary Note 2.

Assuming a polar grid of *P* arcs (arcs are enumerated with the enumeration initiating from tissue surface) and *L* radial lines (see Supplementary Fig. 3b) with a total of *PL* points at positions **r**_*p,l*_, and let the vector **m**_*i*_ =[*m*_i_(**r**_1,1_), *m*_i_(**r**_1,2_), …, *m*_i_(**r**_1,L_), *m*_i_(**r**_2,1_), …, *m*_i_(**r**_*p,l*_), …,*m*_i_(**r**_P,L_)]^T^ correspond to the values of the Eigenfluence parameter *i* (*i*=1, 3) over all such points, the new inverse problem is defined as the minimization of cost function *f*_grid_ defined in equation (6) under the constraints defined in equation (7).


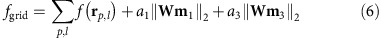



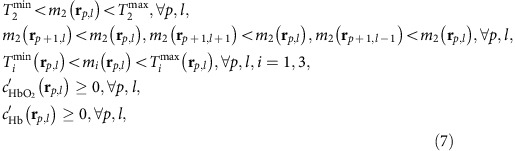


In equation (6), the term 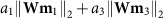
implements the spatial smoothness constraints imposed on *m*_1_(**r**_*p,l*_) and *m*_3_(**r**_*p,l*_). The matrix **W** describes a connectivity graph defined on the grid of points assumed (Supplementary Fig. 3b) and its structure and function are analytically described in Supplementary Note 2. In equation (7) 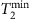
and 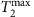 are the search-space limits for *m*_2_(**r**_*p,l*_), which are constant for all grid points and correspond to the maximum and minimum values of *m*_2_ on the training data set (Fig. 1g). 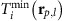
and 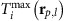, with *i*=1,3 are the search-space limits for *m*_1_(**r**_*p,l*_) and *m*_3_(**r**_*p,l*_), which are computed per grid point in a preprocessing step as described in Supplementary Note 2. The inverse problem defined by equations (6) and (7) was hereby solved through the utilization of the sequential quadratic programming algorithm of MATLAB toolbox.

*Fluence correction and sO_2_ quantification*. The minimization of cost function *f*_grid_ (equation (6)) under the constraints of equation (7) yields an estimate of *m*_*i*_(**r**_*p,l*_) for each Eigenfluence parameter *i* and each grid point **r**_*p,l*_. The Eigenfluence parameters in the convex hull of the grid are in the following estimated by means of cubic interpolation. We note that due to the nature of diffuse light propagation, the Eigenfluence parameters are expected to vary smoothly in tissue and thus their interpolation is not expected to introduce large errors in the result (see Supplementary Note 3 and Supplementary Table 2). The wavelength dependence of light fluence is computed for each pixel within the convex hull of the grid as in *Φ*′(**r,***λ*)=*Φ*_M_(*λ*)+*m*_1_(**r**)*Φ*_1_(*λ*)+*m*_2_(**r**)*Φ*_2_(*λ*)+*m*_3_(**r**)*Φ*_3_(*λ*), where *Φ*_*i*_(*λ*) is the *i*th fluence Eigenspectrum. Finally, a spectrally corrected eMSOT image is obtained after dividing the original image *P*(**r**,*λ*) with the normalized wavelength-dependent light fluence *Φ*′(**r,***λ*) at each position **r** and wavelength *λ*, i.e., *P*^eMSOT^(**r**,*λ*)=*P*(**r**,*λ*)/ *Φ′*(**r,***λ*). The relative concentrations of HbO_2_ and Hb (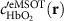, *c′*_Hb_^eMSOT^(**r**)) are computed for each pixel of *P*^eMSOT^(**r**,*λ*) image independently through non-negative constrained least squares fitting with the spectra of oxygenated and deoxygenated haemoglobin. Thus, the eMSOT blood sO_2_ maps retain the original resolution of the MSOT imaging system.

We note that both the Eigenspectra model and the inversion scheme were hereby optimized for the application of small-animal imaging. The Eigenspectra model was trained for a maximum depth of 1 cm and the inversion scheme was designed with respect to the same tissue depth and optical properties within the physiological range (Supplementary Note 2, 3).

### Linear unmixing

Under the simplifying assumption that the light fluence attains a flat spectrum irrespective of the tissue position *Φ*(**r,***λ*)=*Φ*(**r**) and by assuming haemoglobin as the major absorber in tissue, optoacoustic spectra can be modelled as a linear combination of the spectra of oxy- and deoxy-haemoglobin. The term linear unmixing refers hereby to the computation of the relative concentrations of HbO_2_ and Hb (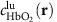, *c*_Hb_^lu^(**r**)) and subsequently blood sO_2_, through non-negative constrained least squares fitting of the original image *P*(**r**,*λ*) with the spectra of Hb and HbO_2_.

### Blood phantom preparation

For validating the accuracy of eMSOT in quantifying blood oxygenation in deep tissue, we prepared tissue mimicking phantoms, containing blood at known oxygenations levels. Specifically, for simulating tissue background, 2-cm-diameter cylindrical solid phantoms were created by using 1.5% Agarose Type I, Sigma-Aldrich (solidifying in <37°), 2% intralipid and 3–5% freshly extracted pig blood diluted in NaCl. Different blood oxygenation levels were achieved by diluting oxygen in whole blood (oxygenation process) or by mixing the blood with different amounts of sodium dithionite (Na_2_O_4_S_2_) (deoxygenation process)^31^. The blood oxygenation levels were monitored using a Bloodgas Analyser (Eschweiler Gmbh & Co. KG, Kiel Germany).

### Cryoslicing colour imaging and haematoxylin and eosin staining of tumours

After MSOT acquisition, a subset of the mice bearing 4T1 mammarian tumours (*n*=4) were killed and examined for tumour and tissue anatomy. Mice were embedded in an optimal cutting temperature compound (Sakura Finetek Europe BV, Zoeterwonde, NL) and frozen at −80 °C. In the following, the mice were sliced at an orientation similar to the one of MSOT imaging and colour photographs were recorded. The cryoslicing imaging system is based on a cryotome (CM 1,950, Leica Microsystems, Wetzlar, Germany), fitted with CCD-based detection camera. During this process, 10 μm slices throughout the whole tumour volume were collected for further histological analysis.

Several slides per tumour were subjected to haematoxylin and eosin staining and imaging. The slides containing 10 μm cryosections were first pre-fixed in 4% paraformaldehyde (PFA) (Santa Cruz Biotechnology Inc., Dallas, Texas, USA). Then, they were rinsed with distilled water and incubated 30 s with Haemotoxylin acide by Meyer (Carl Roth, Karlsruhe, Germany) to stain the cell nuclei. The slides were then rinsed in tap water again before incubation for 1 s in Eosin G (Carl Roth, Karlsruhe, Germany) to stain cellular cytoplasm. After rinsing in distilled water, the slides were dehydrated in 70%, 94% and 100% ethanol and incubated for 5 min in Xylene (Carl Roth, Karlsruhe, Germany) before being cover slipped with Rotimount (Carl Roth, Karlsruhe, Germany) cover media. Representative slides were observed using Zeiss Axio Imager M2 microscope with AxioCam 105 colour, and pictures were then processed using a motorized stitching Zen Imaging Software (Carl Zeiss Microscopes GmbH, Jena, Germany).

### Pimonidazole staining of tumour tissues

A subset of the tumour-bearing mice (*n*=4) was examined for functional characteristics of the tumours by Pimonidazole histological staining. The hypoxia marker Pimonidazole (Hypoxyprobe, catalogue #HP6-100 kit, Burlington, MA, USA) was injected intraperitonially at 100 mg/kg body weight in a volume of 0.1 ml saline ≈1.5 h before tumour excision, and the perfusion marker Hoechst 33342 (Sigma, Deisenhofen, Germany) was administered intravenous at 15 mg/kg body weight in a volume of 0.1 ml saline 1 min before the tumour-bearing mice were killed. The tumours were excised immediately after the animals were killed. The orientation of the tumours with respect to the mouse body was retained. Eight micrometers cryosections were sliced throughout the tumour. The cryosections were fixed in cold (4 °C) acetone, air dried and rehydrated in PBS before staining. Pimonidazole was stained with the FITC-labelled anti-Pimonidazole antibody (Hypoxyprobe, Burlington, MA, USA) diluted 1:50 in primary antibody diluent (PAD, Serotec, Oxford, UK) by incubating for 1 h at 37 °C in the dark.

### Code and data availability

The code and all relevant data of this work will be made available upon request.

## Additional information

**How to cite this article:** Tzoumas, S. *et al.* Eigenspectra optoacoustic tomography achieves quantitative blood-oxygenation imaging deep in tissues. *Nat. Commun.* 7:12121 doi: 10.1038/ncomms12121 (2016).

## Supplementary Material

Supplementary Information Supplementary Figures 1-8, Supplementary Tables 1 & 2, Supplementary Notes 1-6 and Supplementary References.
