# Supplementary material for: Eigenspectra optoacoustic tomography achieves quantitative blood oxygenation imaging deep in tissues
Source: Nat Commun. 2016 Jun 30;7:12121. doi: 10.1038/ncomms12121 (PMC4931322; doi:10.1038/ncomms12121)
Supplement: Supplementary Information — Supplementary Figures 1-8, Supplementary Tables 1 & 2, Supplementary Notes 1-6 and Supplementary References. [file ncomms12121-s1.pdf]

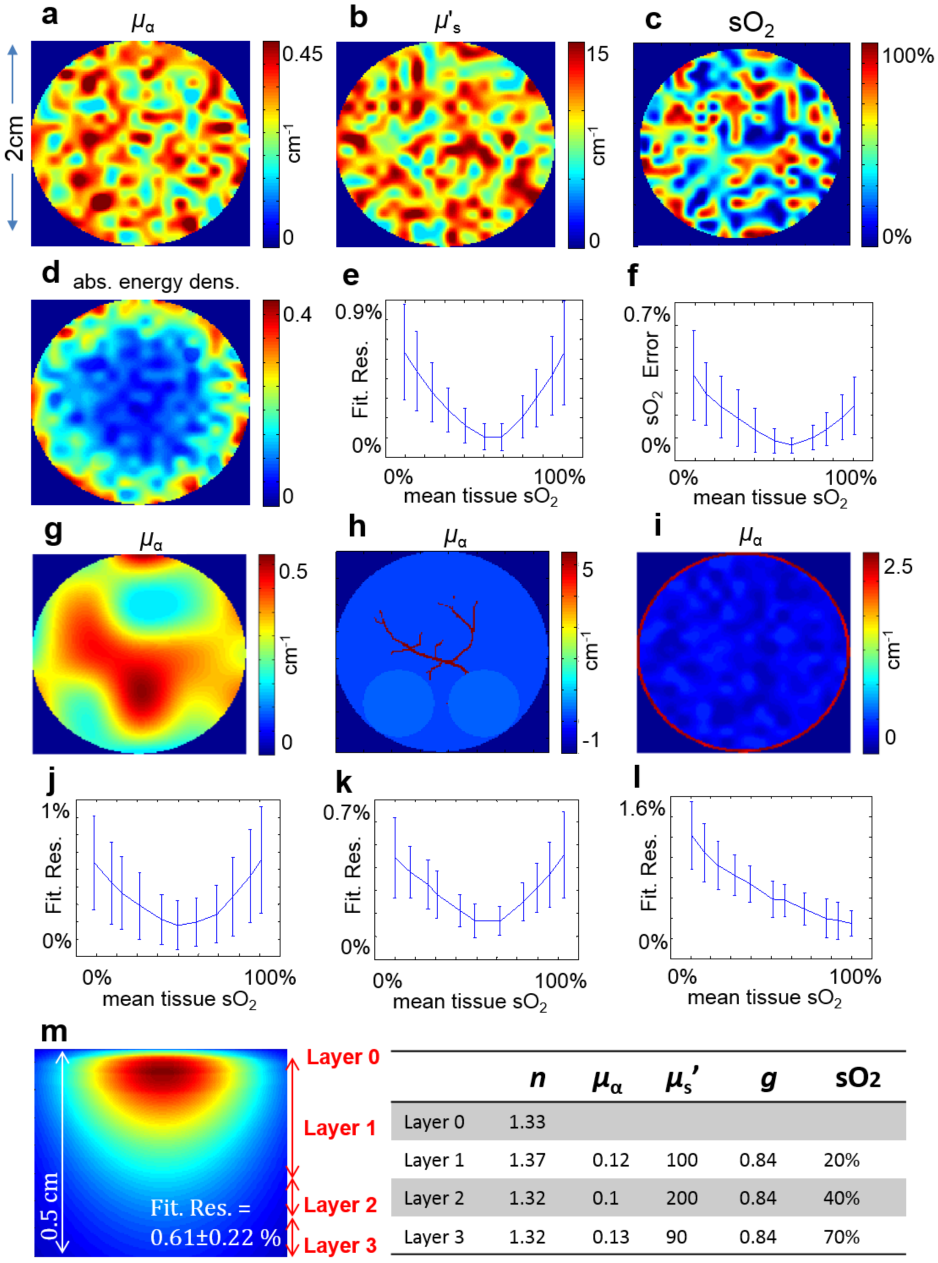

	n	μ_a	μ_s'	g	sO2
Layer 0	1.33				
Layer 1	1.37	0.12	100	0.84	20%
Layer 2	1.32	0.1	200	0.84	40%
Layer 3	1.32	0.13	90	0.84	70%

Supplementary Figure 1. Numerical validation of the *Eigenspectra* model of light fluence in tissue simulations of arbitrary structures. **(a,b)** Examples of the assumed random spatial maps of **(a)** $\mu_a(\mathbf{r})$ at 800 nm and **(b)** $\mu_s'(\mathbf{r})$, with random, normally distributed values. **(c)** Example of a random spatial map of sO_2. **(d)** Example of multi-wavelength absorbed energy density simulation (wavelength 800 nm presented), created using the FEM DE light propagation model. **(e)** Statistics (–error-bars indicate standard deviation) of the fitting residual of the *Eigenspectra* model computed from all pixels of each simulated multispectral dataset. **(f)** Error propagated to sO_2 estimation due to the fluence approximation using the *Eigenspectra* model (forward model error). **(g-i)** Examples of tissue simulations of low spatial variation of optical properties **(g)**, partially uniform optical properties with highly absorbing vessel like structures **(h)** and cases of high melanin absorption at the tissue surface as well as wavelength dependent scattering **(i)**. **(j-l)** Statistics of the fitting residual of the forward model corresponding to the simulations presented in **(g-i)**, respectively. **(m)** Monte Carlo simulations of the wavelength dependent light fluence (fluence of one wavelength is presented) in the ballistic and semi-ballistic regime, assuming semi-uniform multi-layered tissue; Layers are highlighted with red arrows and their optical properties are summarized in the enclosed table. Statistics of the fitting residual of the *Eigenspectra* model (mean and standard deviation) are also presented.

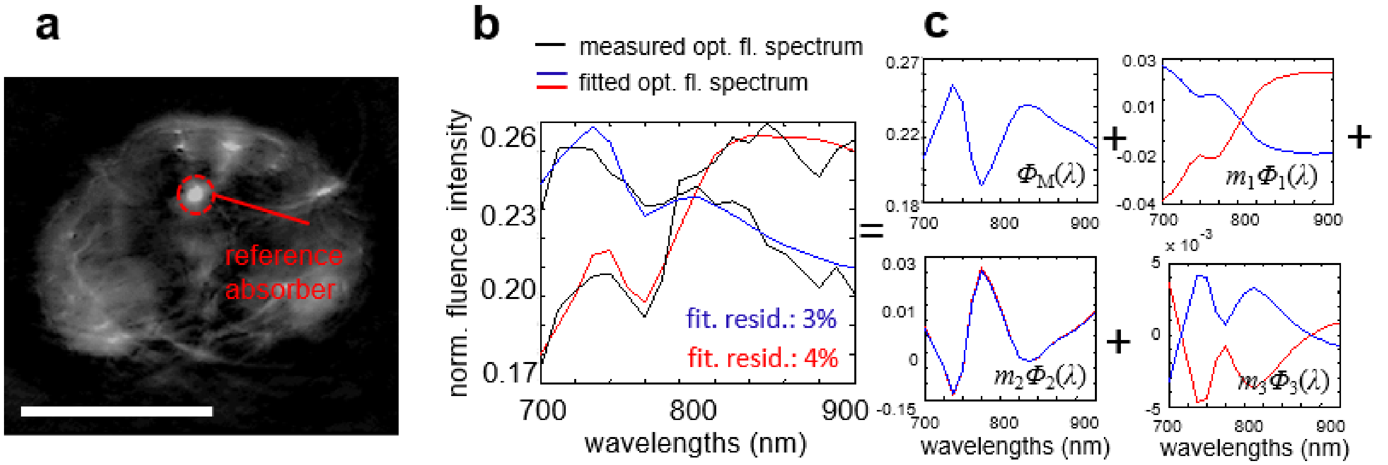

Supplementary Figure 2. Validation of the *Eigenspectra* model using light fluence measurements obtained *in vivo* and *post mortem*. (a) MSOT image (one wavelength presented) of a CD1mouse imaged in the abdominal region with a capillary tube containing a reference absorber inserted in the lower abdominal area (red circle). Scale bar, 1 cm. **(b)** Comparison of the measured spectrum of light fluence in the area of absorber insertion (black curves) with the fitted spectrum using the 3-dimensional *Eigenspectra* model in the case of *in-vivo* imaging (blue curve) and *post-mortem* imaging (red curve). **(c)** The two light fluence spectra corresponding to the *in vivo* (blue) and *post mortem* case (red) are decomposed into a linear combination of spectra $\Phi_M(\lambda)$, $m_1\Phi_1(\lambda)$, $m_2\Phi_2(\lambda)$ and $m_3\Phi_3(\lambda)$.

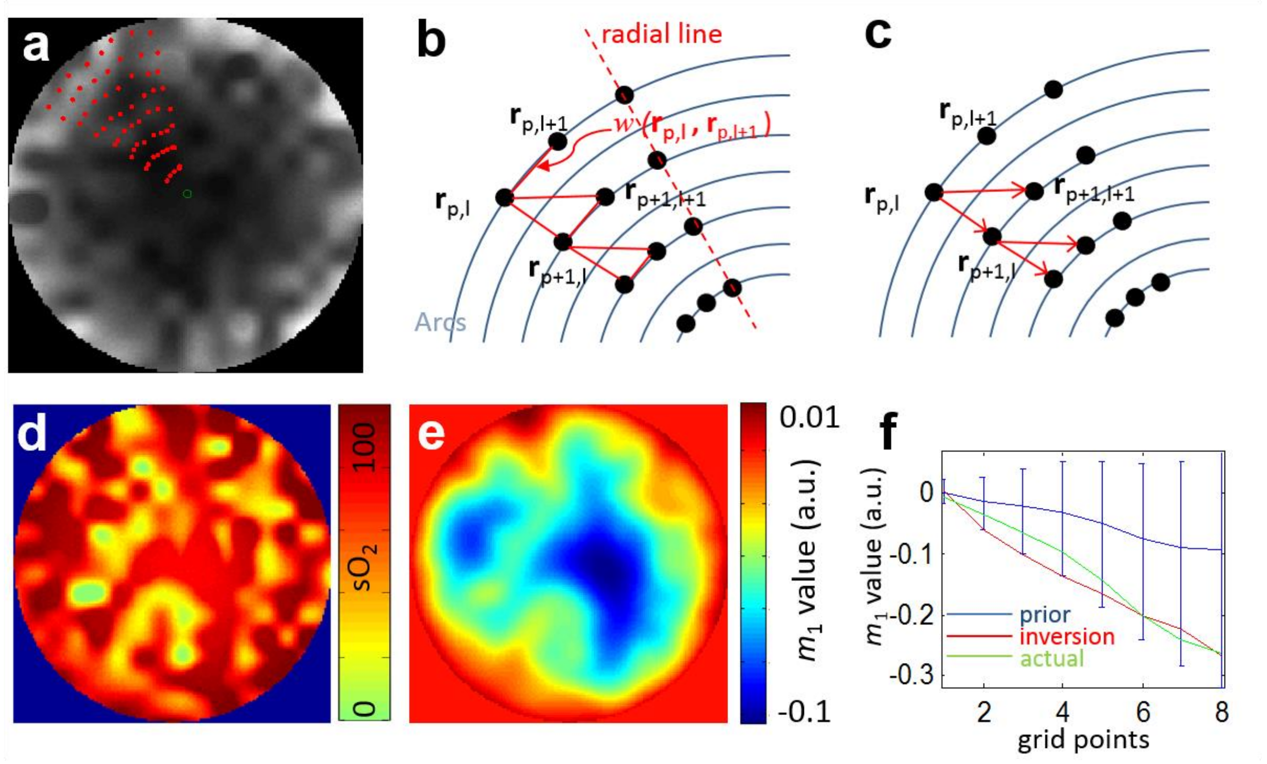

Supplementary Figure 3. Explanation of eMSOT constrained inversion. (**a**) eMSOT inversion is performed simultaneously on a grid of points in the image domain (red points). (**b**) A non-directed weighted connectivity graph defined on the grid of points penalizes large variations of the *Eigenfluence* parameters m_1 and m_3 between neighbor points. The penalization is inversely proportional to the distance w between the grid points. (**c**) A directed graph on the grid of points enforces a decrease on the values of m_2 with depth. (**d-f**) An initial approximation of tissue blood oxygenation is obtained using nonnegative constrained least squares fitting (**d**) and used for obtaining a prior estimate of $\acute{m}_1(\mathbf{r})$ (**e**) and $\acute{m}_3(\mathbf{r})$ model parameters. These prior estimates are used for constraining the total search space for m_1 and m_3 during optimization. (**f**) Prior $\acute{m}_1$ estimate (blue line), limits of the search space (blue vertical lines), actual m_1 values (green line) and m_1 values estimated after optimization (red line) for a radial line of the grid presented in (**a**).

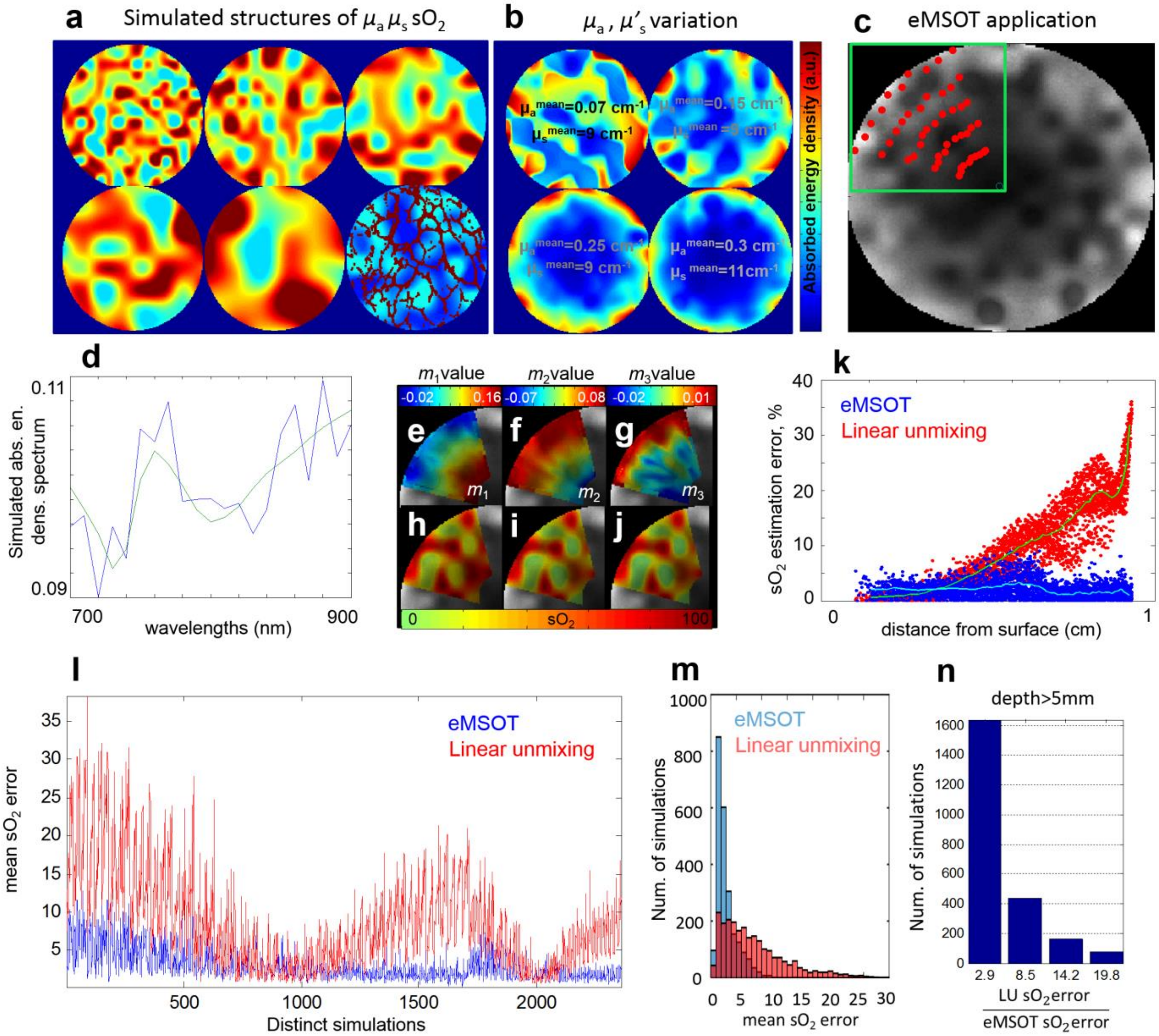

Supplementary Figure 4. Numerical validation of eMSOT in simulations of arbitrarily structured tissues. (a) Examples of the assumed random maps of optical absorption, optical scattering and sO_2 varying from finely granulated to smoothly varying structures and vessel-like patters. The combination of these maps was used to simulate the absorbed energy density of complex tissue using a light propagation model. (**b**) The simulations of multispectral absorbed energy density were formed using varying mean optical properties simulating weakly to strongly absorbing/scattering tissue. **(c)** Simulated multispectral optoacoustic image (one wavelength presented). A polar grid is placed on the upper left part of the image for analysis using eMSOT. **(d)** Original (green) and noisy (blue) simulated absorbed energy density spectrum stemming from one pixel of **(c)**. **(e-g)** Maps of *Eigenfluence* parameters m_1, m_2 and m_3, respectively, obtained after inversion and interpolation. **(h-i)** sO_2 estimation using linear unmixing (**h**) and eMSOT (**i**). (**j**) Actual simulated sO_2 map. (**k**) sO_2 estimation error corresponding to all pixels of the analyzed area using conventional linear unmixing (red points) and eMSOT (blue points), sorted per depth. (**l**) Mean sO_2 error of linear unmixing (red) and eMSOT (blue) corresponding to each simulated data-set tested (2358 data-sets in total). (**m**) Histogram of the mean sO_2 estimation error corresponding to eMSOT (blue) and linear unmixing (red) for all simulated data-sets tested. (**n**) Histogram of the relative sO_2 estimation error of linear unmixing as compared to eMSOT for all simulated data-sets tested and simulated tissue depths > 5mm.

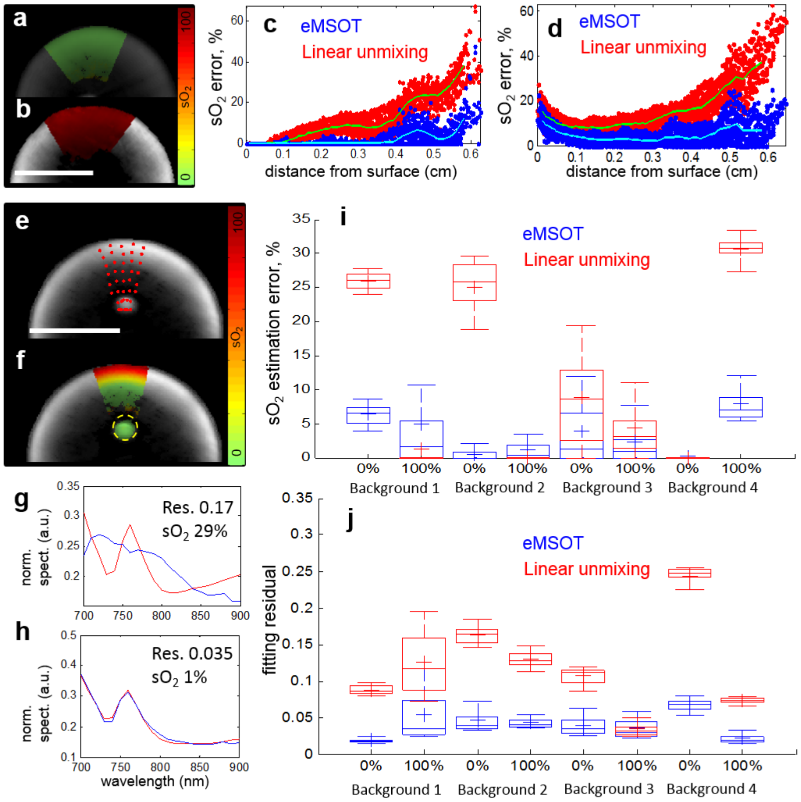

Supplementary Figure 5. Validation of eMSOT using blood phantoms. (**a, b**) eMSOT sO_2 estimation in the case of a uniformly deoxygenated blood phantom (**a**) and a uniformly oxygenated phantom (**b**). Scale bar, 1 cm. (**c, d**) sO_2 estimation error of eMSOT (blue dots) and linear unmxing (red dots) sorted per depth for the case of the deoxygenated phantom (**c**) and oxygenated phantom (**d**). (**e, f**) eMSOT grid application (**e**) and sO_2 estimation (**f**) in the case of a blood phantom with non-uniform background oxygenation containing an insertion of 0% sO_2. The insertion area is marked with a yellow dashed circle. Scale bar, 1 cm. (**g, h**) Spectral fitting and sO_2 estimation corresponding to a pixel in the insertion area in (**f**) using linear unmixing (**g**) and eMSOT (**h**). The blue curves correspond to $P(\mathbf{r},\lambda)$ (**g**) and $P^{eMSOT}(\mathbf{r},\lambda)$ (**h**) while the red curves correspond to $c_{HbO2}{}^{lu}(\mathbf{r})\varepsilon_{HbO2}(\lambda)+c_{Hb}{}^{lu}(\mathbf{r})\varepsilon_{Hb}(\lambda)$ (**g**) and $c'_{HbO2}{}^{eMSOT}(\mathbf{r})\varepsilon_{HbO2}(\lambda)+c'_{Hb}{}^{eMSOT}(\mathbf{r})\varepsilon_{Hb}(\lambda)$ (**h**). (**i**) Statistics on the sO_2 estimation error of eMSOT (blue) and linear unmixing (red) corresponding to the insertion region of eight different phantoms of four different backgrounds each containing an insertion of 0% and 100% sO_2. (**j**) Statistics on the fitting residual of eMSOT (blue) and linear unmixing (red) corresponding to the insertion region. Statistics in (**i, j**) are derived from all pixels the ROIs corresponding to the insertion area of each phantom. The boxes include 25%-75% and the error-bars 9%-91% of the data. The mean value is denoted with the plus symbol.

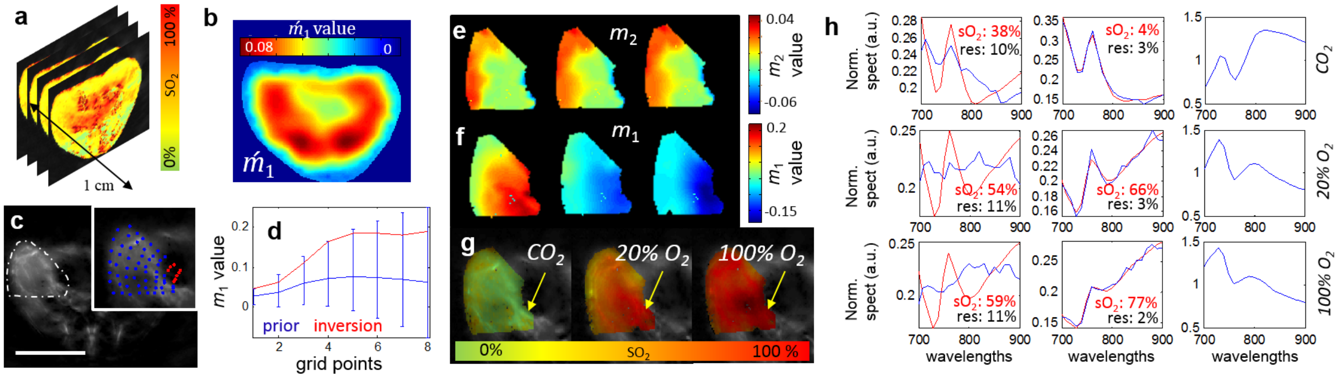

Supplementary Figure 6. Explanation of eMSOT application on experimental tissue images. (**a**) Initial sO_2 maps (computed using linear unmixing) corresponding to multiple MSOT slices surrounding the central slice to be analyzed. (**b**) Prior $\acute{m}_1(\mathbf{r})$ map computed using a 3D FEM DE light propagation model and the initial sO_2 maps as described in **Supplementary Note 2**. (**c**) Selection of a high intensity area in a well-reconstructed part of the image for the automatic application of a grid for eMSOT application. Scale bar, 1 cm. (**d**) Prior $\acute{m}_1$ (blue line), limits of search space (blue vertical lines) and estimated m_1 after eMSOT inversion, corresponding to a radial line of the grid in (**c**). (**e-g**) m_2 (**e**), m_1 (**f**) and sO_2 maps (**g**) computed after eMSOT inversion for the same tissue area under three different breathing conditions. (**h**) Original optoacoustic spectra ($P(\mathbf{r},\lambda)$; left, blue), eMSOT spectra ($P^{eMSOT}(\mathbf{r},\lambda)$; middle, blue) and estimated spectrum of light fluence (right) corresponding to a deep tissue point (yellow arrow in **g**). Red curves correspond to $c_{HbO2}{}^{lu}(\mathbf{r})\varepsilon_{HbO2}(\lambda)+c_{Hb}{}^{lu}(\mathbf{r})\varepsilon_{Hb}(\lambda)$ (left) and $c'_{HbO2}{}^{eMSOT}(\mathbf{r})\varepsilon_{HbO2}(\lambda)+c'_{Hb}{}^{eMSOT}(\mathbf{r})\varepsilon_{Hb}(\lambda)$ (middle). The fitting residual and the estimated sO_2 value are also presented in each case.

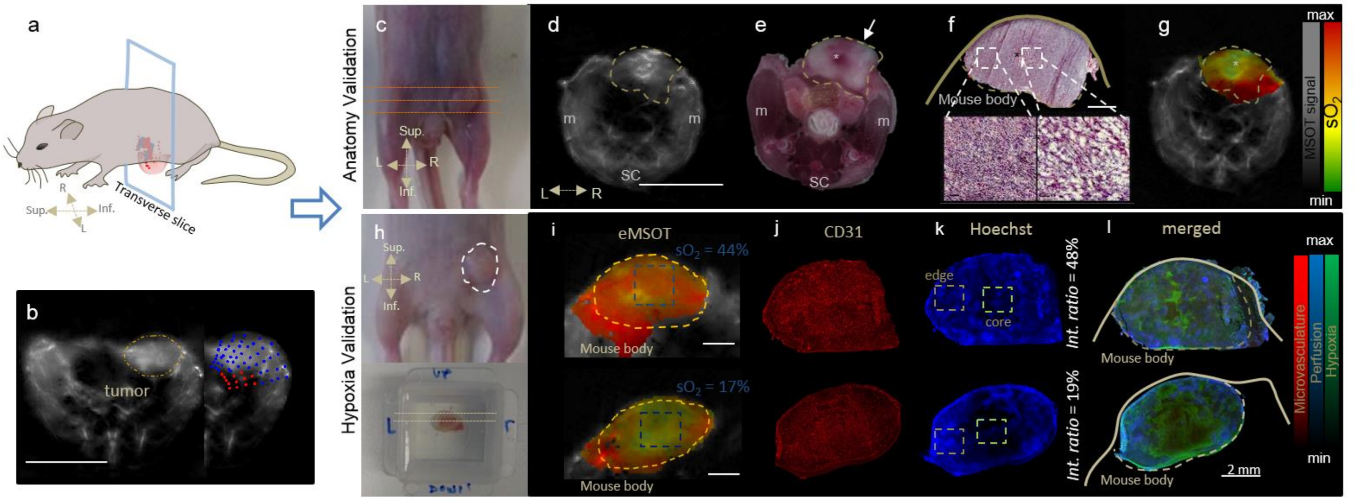

Supplementary Figure 7. eMSOT tumor imaging and histological validation. (**a**) Schematic representation of MSOT imaging at a transverse slice within the tumor area (**b**) Cross-sectional optoacoustic image at a central tumor transverse slice. The tumor region is segmented with a dashed line. The eMSOT grid is further presented (blue and red dots). (**c**) Image of the lower abdominal area displaying the orthotopic mammary tumor. Dashed lines present the orientation of cryoslicing and MSOT imaging. (**d-g**) Anatomical optoacoustic image (**d;** Scale bar, 1cm, m: muscle, sc: spinal cord) and the corresponding cryosliced color photography (**e**), H&E staining of the tumor region (**f;** Scale bar, 2mm) and eMSOT sO_2 analysis of the tumor area (**g**). (**h,** lower) Excised tumor used for functional staining. Yellow dashed lines indicate the slicing orientation. (**i-l**) Examples of a highly perfused (upper row) and low perfused (lower row) tumor analysed with eMSOT for sO_2 estimation (**i**), CD31 staining (**j**), Hoeachst33342 staining (**k**), and merged with Pimonidazole staining (**l**). Scale bar, 2mm. The tumor margins are presented in (**i**) indicated by yellow dashed lines. Blue dashed rectangles indicate a region in the tumor core, the average sO_2 values of which is displayed on the upper right. The intensity ratio of Hoechst33342 staining was calculated by dividing the mean intensity value in the tumor core (green dashed rectangle in (**k**)) over the one in the tumor boundary (grey rectangle in (**k**)).

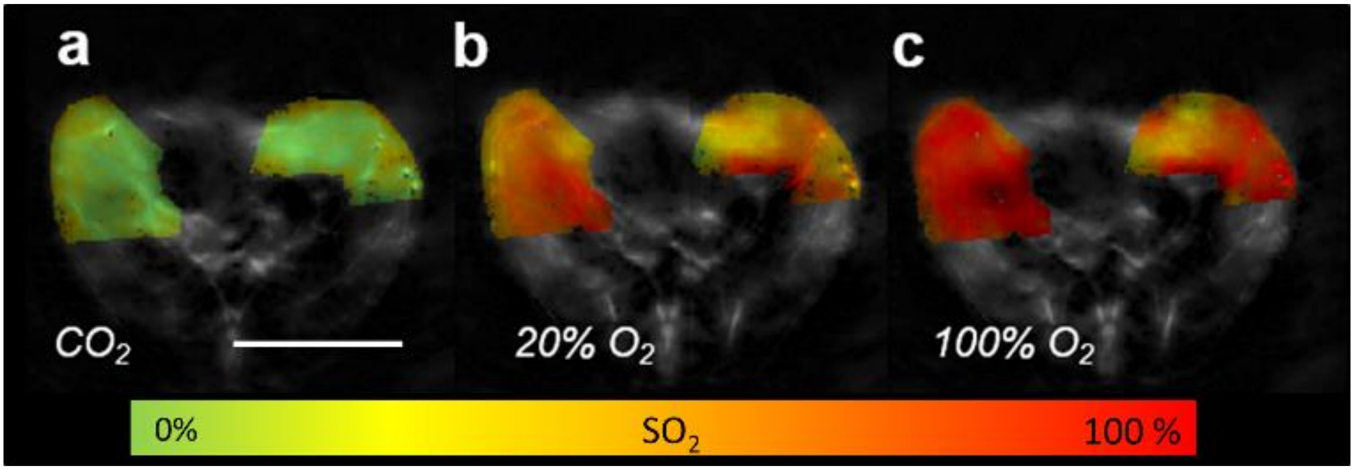

Supplementary Figure 8. Comparison of healthy tissue and tumor sO_2 measurements under a breathing challenge. (a-c) Healthy tissue (left) and tumor (right) sO_2 estimation post-mortem after CO_2 breathing (**a**) and *in-vivo* under 20% O_2 (**b**) and 100% O_2 breathing (**c**).

	Physiological range (30%-80% mean sO_2)				0%-30% mean sO_2	80%-100% mean sO_2	Vessel network (30%-80% sO_2)	
μ_α^{mean} (cm^{-1})	[0.07-0.15]		[0.2-0.3]		[0.07-0.3]	[0.07-0.3]	[0.1, 0.2, 0.3]	
μ_s^{mean}(cm^{-1})	[7-11]		[7-11]		[7-11]	[7-11]	[7, 9, 11]	
Noise lvl.	2.5%	4.5%	2.5%	4.5%	2.5%	2.5%	2.5%	2.5%
Scale							1-3	3-6
Mean sO_2 error	2.36% (4.54%)	2.67% (4.65%)	2.82% (7.9%)	3.38% (7.9%)	5.1% (15.6%)	1.85% (11%)	2.45% (5.83%)	2.0% (4.4%)
% of pixels <10% error	98.6% (89.4%)	98.1% (89.1%)	97.1% (70.8%)	95.0% (70.4%)	85.8% (38%)	99.5% (56%)	98.3% (81.7%)	99.1% (87.5%)
% of pixels <15% error	99.8% (97.2%)	99.7% (97%)	99.3% (85%)	98.7% (84.8%)	97% (57%)	99.9% (74.9%)	99.8% (93%)	99.8% (96%)

Supplementary Table 1. Statistics of the eMSOT performance as evaluated on a large simulated data-set composed of 2358 distinct simulations (red corresponds to conventional linear unmixing).

Grid points	12	30	56	108
Av. computational speed (sec)	1.8 sec	10 sec	52 sec	487 sec
Mean sO_2 error	3.16%	2.74%	2.5%	2.36%
% of pixels <10% error	95.9%	97.7%	98.1%	98.5%

Supplementary Table 2. Statistics of the eMSOT performance as a function of grid density. Statistics correspond to 108 simulated data-sets of $\mu_\alpha^{mean} \in [0.1\text{-}0.3]\ cm^{-1}$, $\mu_s^{mean}=10cm^{-1}$ and mean sO_2 varying between 30%-80%.

Supplementary Note 1: Numerical and experimental validation of the *Eigenspectra* model of light fluence (forward model validation).

For validating the accuracy of the *Eigenspectra* model for light fluence ($\Phi_M(\lambda)$, $\Phi_1(\lambda)$, $\Phi_2(\lambda)$, $\Phi_3(\lambda)$) over light fluence spectra created in arbitrary tissues, we created simulations of the absorbed energy density of arbitrary tissues at different wavelengths (700 nm to 900 nm with a step of 10 nm), using light propagation models. Assuming a circular structure of 1 cm radius, random maps of optical absorption [$\mu_\alpha(\mathbf{r})$] and reduced scattering coefficient [$\mu_s'(\mathbf{r})$] were formed (**Supplementary Fig. 1a** and **b**, respectively), the values of which follow a Gaussian distribution ($\mu_\alpha(\mathbf{r}) \sim N(\mu_\alpha^{mean}, \mu_\alpha^{std})$ where $\mu_\alpha^{mean} \in\{0.07, 0.1, 0.15, 0.2, 0.25, 0.3, 0.35\}$ cm^{-1} and μ_α^{std}=0.1 cm^{-1}, $\mu_s'(\mathbf{r}) \sim N(\mu_s^{mean}, \mu_s^{std})$ where $\mu_s^{mean}\in\{7, 9, 11\}$ cm^{-1} and μ_s^{std}=3 cm^{-1}. The so created absorption maps ($\mu_\alpha(\mathbf{r})$) correspond to tissue absorption at an excitation wavelength of 800 nm (isosbestic point of hemoglobin). The absorption maps for different excitation wavelengths are computed based on the one at 800 nm and the absorption spectra of oxy- and deoxy-hemoglobin. The relative amount of oxy- versus deoxy-hemoglobin at each position **r** is defined by a random map of tissue blood oxygenation (**Supplementary Fig. 1c**). Different blood sO_2 maps were simulated (one example presented in **Supplementary Fig. 1c**) with spatially varying random oxygenation values, and with an average tissue oxygenation varying from ~10% to 90% and a standard deviation of 30%.

The multispectral absorption and scattering maps were employed in a 2D finite-element-method (FEM) solution of the diffusion equation (DE)[1] to simulate multispectral optoacoustic data-sets (i.e. multi-wavelength absorbed energy density) of tissue with arbitrary structure, optical properties and oxygenation. One such example is shown in **Supplementary Fig. 1d** for a single wavelength. From these datasets, the normalized wavelength dependent light fluence $\Phi'(\mathbf{r},\lambda)=\Phi(\mathbf{r},\lambda)/\|\mathbf{\Phi(r)}\|_2$ was calculated for each position **r** in the image. The residual value obtained after comparing the simulated fluence spectra $\mathbf{\Phi'(r)}$ to their approximation using the basis functions of the *Eigenspectra* model ($\mathbf{\Phi'_{Model}(r)}$) was computed (res $=\|\mathbf{\Phi'(r)}-\mathbf{\Phi'_{Model}(r)}\|_2/\|\mathbf{\Phi'(r)}\|_2$) for each pixel in the image **r** and statistics of this residual value are presented in **Supplementary Fig. 1e.** Statistics correspond to all pixels of 21 simulations per mean oxygenation level, corresponding to different mean optical absorption and scattering (231 simulations in total). **Supplementary Fig. 1f** further plots the error of the forward model in the sO_2 estimation (i.e. the error propagated in sO_2 estimation due to the approximation of $\Phi'(\mathbf{r},\lambda)$ with $\Phi'_{Model}(\mathbf{r},\lambda)$).

The *Eigenspectra* forward model was tested with 231 simulations of high (**Supplementary Fig. 1a-c**) and 231 simulations of low spatial variation of optical properties (example shown in **Supplementary Fig. 1g**) and blood oxygenation maps. Moreover the forward model was tested in simulations of blob-like features (representing organs) and vessel-like structures (**Supplementary Fig. 1h**). In this case, the blob-like structures correspond to μ_α = 0.3 cm^{-1}, the background to μ_α = 0.1 cm^{-1} and the vessel like structures to μ_α = 5.4 cm^{-1} and μ_s' = 16 cm^{-1}. The μ_s' and sO_2 maps corresponding to the background followed a random distribution as previously described and the sO_2 of the vessel-like structure was retained uniform and 25% higher than the mean oxygenation of the background. Statistics on the fitting residual of the forward model on the simulations of **Supplementary Fig. 1a, g, h** are presented in **Supplementary Fig. 1e, j, k**, respectively. We observed a small error in the forward model independently of tissue structure and the variations of optical properties and tissue oxygenation.

To assess the potential influence of parameters not included in the model such as the absorption of melanin and the wavelength dependence of scattering we further created simulations containing a strongly absorbing melanin component at the tissue surface (μ_α = 2.5 cm^{-1}) and an exponentially decaying scattering coefficient (μ_s'= $18.9(\lambda/500)^{-0.6}$ cm^{-1}) that corresponds to whole blood measurements[2]; an example presented in **Supplementary Fig. 1i**. The assumed optical properties were again following a normal distribution with $\mu_\alpha(\mathbf{r}) \sim N(\mu_\alpha^{mean}, \mu_\alpha^{std})$ where $\mu_\alpha^{mean} \in\{0.07, 0.1, 0.15, 0.2, 0.25, 0.3, 0.35\}cm^{-1}$ and μ_α^{std}=0.1 cm^{-1}, $\mu_s'(\mathbf{r}) \sim N(\mu_s^{mean}, \mu_s^{std})$) where $\mu_s^{mean} \in\{7, 9, 11\}$ and μ_s^{std} =3 cm^{-1} (21 simulations per mean oxygenation, 231 simulations in total). Similar to the absorption maps, the so created scattering maps $\mu_s'(\mathbf{r})$ correspond to tissue scattering at an excitation wavelength of 800 nm. The scattering maps for different excitation wavelengths are computed based on the one at 800 nm and the exponentially decaying curve of the scattering coefficient. In this case the fitting residual of the forward model is increased (**Supplementary Fig. 1 l**) but is still preserved in relatively low levels indicating that the model retains accuracy despite the simplifying assumptions in its creation.

The accuracy of the forward model in the ballistic regime was tested using Monte Carlo simulations[3] of multi-layered tissue (**Supplementary Fig. 1 m**). Four different tissue layers were assumed with different oxygenation levels and optical properties. In this case the fitting residual of the forward model is similar to the one when using the diffusion equation: 0.61±0.22%.

The graphs indicate a small model error, supporting the hypothesis that a simple affine model with only three *Eigenspectra* can capture the spectral variability of $\Phi'(\mathbf{r},\lambda)$ in complex tissue structures, independently of the distribution of the optical properties. We hereby note that the error in sO_2 estimation depicted in **Supplementary Fig. 1f** is just indicative of the model accuracy (error of the forward model) and does not relate to the actual blood sO_2 estimates that can be obtained through this procedure by solving the inverse problem (estimation error of the inverse problem).

To experimentally investigate the validity of the *Eigenspectra* model of light fluence we obtained measurements from small animals *in-vivo* and *post-mortem*. We measured the light fluence in tissue by inserting a reference chromophore with well characterized spectrum within tissue. Specifically, a capillary tube was rectally inserted into an anesthetized CD1 mouse and the animal was imaged in the lower abdominal area *in-vivo* using the MSOT system. The capillary tube was filled with black India ink, the spectrum of which was previously measured in the photospectrometer. The animal was imaged *in-vivo* under 100% O_2 breathing and *ex-vivo*. These two different physiological conditions were employed in order to investigate the influence of the average background tissue oxygenation on the spectrum of the light fluence.

The per-wavelength image intensity at the region of the ink insertion (i.e. the optoacoustic measured spectrum which corresponds to the multiplication of the local absorption with the local light fluence) was elementwise divided by the actual absorption spectrum of ink. The resulting spectrum after division corresponds to the wavelength dependence of the local light fluence. The measured light fluence spectrum computed in this way was fitted to the *Eigenspectra* model and the two curves and the fitting residual are presented in **Supplementary Fig. 2.**

Supplementary Fig. 2a presents a single wavelength optoacoustic image of the mouse in the abdominal area. The area where the light fluence is measured is indicated with a red circle. **Supplementary Fig. 2b** presents the spectrum of the experimentally measured light fluence (black curves) and the fitting result using the *Eigenspectra* model in the case of *in-vivo* (blue curve) and *post-mortem* imaging (red curve). The low fitting residuals indicate good agreement of the model with experimental reality. **Supplementary Fig. 2c** presents the decomposition of the two fitted light fluence spectra as a linear combination of the mean fluence spectrum and the three *Eigenspectra*. While the first and the third *Eigenspectra* components change dramatically with respect to the two different tissue oxygenation states, the second component that corresponds to tissue depth remains relatively unchanged. Moreover the values of the m_1 parameter obtained after fitting were positive in the *post-mortem* case and negative in the *in vivo* case, an observation that is in accordance with the dependence of m_1 on background tissue oxygenation, presented in **Figure 1f**. This observation was confirmed by performing the same experiment in 2 more animals. Overall, the low fitting residual even in the case of experimental data obtained *in-vivo* indicates good agreement between theory and experimental reality.

Supplementary Note 2: Constrained inversion

Spatial smoothness constraint. The spatial characteristics of light fluence were exploited for overcoming the ill-posed nature of the optimization problem defined by Eq. (5). In contrary to tissue absorption which can vary arbitrarily, the light fluence is bound to vary smoothly in space due to the nature of diffuse light propagation. In the context of the *Eigenspectra* model inversion, such *a priori* information can be incorporated by attempting simultaneous inversion on a grid of points defined in the image domain (an example of such a grid is shown in **Supplementary Fig. 3a**).

In our implementation, this *ad hoc* spatial smoothness constraint is enforced by assuming a weighted non-directed graph (**Supplementary Fig. 3b**) that connects the neighbor grid points with edges that carry weights w, which are inverse proportional to the distance between the neighbor grid points. As described in **Methods**, a circular grid of P arcs and L radial lines (**Supplementary Fig. 3b**) is assumed containing PL points at positions $\mathbf{r}_{p,l}$. The arcs are enumerated from 1 to P, with higher indexes indicating larger tissue depths. We define the vector $\mathbf{m_i} = [m_i(\mathbf{r}_{1,1}), m_i(\mathbf{r}_{1,2}), \ldots, m_i(\mathbf{r}_{1,\mathrm{L}}), m_i(\mathbf{r}_{2,1}), \ldots, m_i(\mathbf{r}_{p,l}), \ldots, m_i(\mathbf{r}_{\mathrm{P,L}})]^{\mathrm{T}}$ (dimensions $PL\times1$) which contains all values of the *Eigenfluence* parameter i (i=1…3) over all grid points. Using this notation the objective function of the eMSOT inverse problem is defined in Eq. (6), where the term $a_1 \| \mathbf{Wm}_1 \|_2 + a_3 \| \mathbf{Wm}_3 \|_2$ enforces the *ad hoc* spatial smoothness constraints imposed on m_1 and m_3.

The matrix **W** implements the weighted non-directed connectivity graph (**Supplementary Fig. 3b**) and it has dimensions of $K\times PL$, where K is the total number of edges of the connectivity graph: $K=|E|$ where E is the set of all edges of the connectivity graph**.** We re-enumerate grid points as follows: $\mathbf{r}_{p,l} \rightarrow \mathbf{r}_{(p\text{-}1)P+l}$, so that every grid point has a corresponding index in the range of 1… PL. Let u and v be the indices specifying two of the grid points: $\mathbf{r}_u$ and $\mathbf{r}_v$. If these points are connected, the pair $e_{u,v} = (\mathbf{r}_u, \mathbf{r}_v)$ is an edge, i.e. $e_{u,v} \in E$. Let us now enumerate the edges of the graph and let $k_{u,v} \in \{1 \ldots K\}$ be the index of an edge $e_{u,v}$. Each row of matrix **W** corresponds to a single edge of the connectivity graph shown in **Supplementary Fig. 3b**, and it contains only two non-zero elements corresponding to the points it connects. For instance, for an edge $e_{u,v}$ there is a corresponding row in **W** with an index $k_{u,v}$ that has non-zero elements at the columns u and v corresponding to the connected points $\mathbf{r}_u$ and $\mathbf{r}_v$. The values of the two non-zero elements per row are: $\mathbf{W}_{k_{u,v},u} = d(e_{u,v})$ and $\mathbf{W}_{k_{u,v},v} = -d(e_{u,v})$, where $d(e_{u,v}) = 1/\|\mathbf{r}_u - \mathbf{r}_v\|_2$. The multiplication $\mathbf{Wm}_i$ results into a vector of K elements (corresponding to the K edges), which are equal to $d(e_{u,v})(m_i(\mathbf{r}_u) - m_i(\mathbf{r}_v))$. When the regularization terms $\|\mathbf{Wm}_i\|_2 = \sqrt{\sum_{e_{u,v}\in E} d^2(e_{u,v})(m_i(\mathbf{r}_u) - m_i(\mathbf{r}_v))^2}$, i=1,3, are added to the minimization function f_{grid}, they enforce a simultaneous minimization of the spatial variation of m_1 and m_3 *Eigenfluence* parameters.

The values of the regularization parameters α_1 and α_3 were selected using cross-validation on simulated data-sets with finely granulated structures (**Supplementary Fig. 1a-c**). We did not observe high sensitivity of the result obtained to small changes of the regularization parameters. The same values for the regularization parameters were used for all simulated and experimental data presented in the work.

Constraint of m_2 with tissue depth. An additional spatial fluence constraint is applied in the case of the second *Eigenfluence* parameter m_2. Through simulations of uniform optical properties as well as simulations with randomly varying optical properties it was observed that the values of m_2 are strongly and consistently associated with tissue depth, obtaining lower values in deeper tissue areas. Through the definition of an additional directed graph based on the assumed gird (**Supplementary Fig. 3c**) the value of m_2 at a certain grid point was enforced to obtain larger values than the ones of its direct neighbors placed deeper in tissue. Since the grid arcs are enumerated from 1 to P, with higher indexes indicating larger tissue depths this constraint is expressed through the following set of inequalities:

$$m_2(\mathbf{r}_{p+1,l}) < m_2(\mathbf{r}_{p,l}), m_2(\mathbf{r}_{p+1,l+1}) < m_2(\mathbf{r}_{p,l}), m_2(\mathbf{r}_{p+1,l-1}) < m_2(\mathbf{r}_{p,l}), \forall p, l,$$

Search-space constraints through an initial sO_2 approximation. For further enhancing the inversion stability, additional constraints were imposed to the *Eigenfluence* parameters that relate to both depth and background tissue oxygenation (i.e. m_1 and m_3) based on a first approximate estimate of tissue blood oxygenation. By performing linear spectral unmixing on the raw multispectral optoacoustic images $P(\mathbf{r},\lambda)$ a first estimation map of blood sO_2 levels can be obtained. It is noted that this sO_2 map is incrementally erroneous with tissue depth, however it can serve as a first approximation for constraining the total search-space for m_1 and m_3 to a more relevant sub-space. Using the so created sO_2 map (**Supplementary Fig. 3d**) and by assuming uniform tissue optical properties (i.e. μ_α= 0.3 cm^{-1} at 800 nm and μ_s'=10 cm^{-1}) a light fluence map is simulated using a FEM of the DE. By fitting the simulated light fluence spectra $\Phi'(\mathbf{r},\lambda)$ to the *Eigenspectra* model, prior estimates of all model parameters $\acute{m}_1(\mathbf{r})$, $\acute{m}_2(\mathbf{r})$ and $\acute{m}_3(\mathbf{r})$ can be obtained for each pixel **r**. A map of $\acute{m}_1(\mathbf{r})$ corresponding to the sO_2 map of **Supplementary Fig. 3d** is presented in **Supplementary Fig. 3e** while the values of $\acute{m}_1(\mathbf{r}_{p,l})$ for all grid positions $\mathbf{r}_{p,l}$ corresponding to one radial line of the gird in **Supplementary Fig. 3a** are presented in **Supplementary Fig. 3f** (blue line).

The optimization problem of Eq. (6) is solved, with the values of $m_1(\mathbf{r}_{p,l})$ and $m_3(\mathbf{r}_{p,l})$ constrained to lie within a region surrounding the initial prior estimate $\acute{m}_i(\mathbf{r}_{p,l})$ (blue vertical lines in **Supplementary Fig. 3f**):

$$T_i^{\min}(\mathbf{r}_{p,l}) < m_i(\mathbf{r}_{p,l}) < T_i^{\max}(\mathbf{r}_{p,l}), \forall p, l, i = 1,3.$$

The limits of the allowed search space ($T_i^{\min}(\mathbf{r}_{p,l})$, $T_i^{\max}(\mathbf{r}_{p,l})$) were selected *ad hoc* as a function of the prior *Eigenfluence* values $\acute{m}_i(\mathbf{r}_{p,l})$ and tissue depth, through the comparison of the prior and the real *Eigenfluence* parameters computed in tissue simulations of varying (uniform) optical properties ($\mu_\alpha \in$ [0.1-0.3]cm^{-1} at 800 nm, μ_s'=10 cm^{-1}) and all uniform oxygenation levels. It is noted that the allowed search space is incrementally larger with tissue depth since in deep tissue the original sO_2 estimates (and thus the *Eigenfluence* priors) usually deviate significantly from the true values. **Supplementary Fig. 3f** presents an example of constrained inversion corresponding to a radial grid line of the simulation of **Supplementary Fig. 3a**: The blue line indicates the prior $\acute{m}_1(\mathbf{r}_{p,l})$ across a radial line of the grid, the blue vertical lines indicate the limits of search space, the green line indicates the actual $m_1(\mathbf{r}_{p,l})$ values of the grid points and the red line the estimated ones after nonlinear optimization. The same function for computing the limits ($T_i^{\min}(\mathbf{r}_{p,l})$, $T_i^{\max}(\mathbf{r}_{p,l})$) as a function of the prior $\acute{m}_i(\mathbf{r}_{p,l})$ estimate and tissue depth was used for all simulated and experimental data presented in the work. We note that this constraint (identified through trends in uniform tissue data) may not always be exact in data of complex structures of optical properties and oxygenation; thus excluding in certain cases the optimal solution from the allowed search space. Despite this, the evaluation of **Supplementary Note 4** indicated that the enforcement of this constraint typically leads to a solution close to the optimal one even in such cases, while it minimizes the possibility or an irrelevant convergence in all cases; sacrificing thus accuracy for robustness.

Supplementary Note 3: Numerical validation of eMSOT.

For investigating the ability of eMSOT to obtain accurate quantitative estimates of tissue blood oxygenation we validated its performance using numerical simulations of multi-wavelength absorbed energy density. The absorbed energy density simulations were formed as described in **Supplementary Note 1** using random or semi-random maps of absorption, scattering coefficient and blood oxygenation. A large validation data-set of 2358 different simulations was employed. The optical properties and sO_2 maps followed a random spatial variation with different structural characteristics ranging from finely granulated to smoothly varying structures (**Supplementary Fig. 4a**) as well as highly absorbing vascular structures with an absorption coefficient ranging from 1 to 6 times larger than the mean tissue background (**Supplementary Fig. 4a right low**). In each case the mean tissue optical properties varied from low to high tissue absorption and scattering (**Supplementary Fig. 4b**) in the physiological range ($\mu_\alpha^{mean} \in$ {0.07, 0.1, 0.15, 0.2, 0.25, 0.3} cm^{-1} at 800 nm and $\mu_s^{mean} \in$ {7, 9, 11} cm^{-1}). For each combination of μ_α^{mean}, μ_s^{mean}, different random blood sO_2 maps were assumed ranging from a mean tissue oxygenation of 10% to 90%. Random Gaussian noise with energy varying from 2.5% to 4.5% of the original energy of the spectra in each pixel was further superimposed.

Supplementary Fig. 4c presents a simulated multispectral optoacoustic image (one wavelength presented) after incorporating the optical property maps in a FEM solution of the diffusion equation. A polar grid of 50 points is applied in the upper-left part of the simulation for the application of the eMSOT method. The parameters of inversion and the constraints employed were the same with the ones used for analyzing the *in-vivo* datasets and are analytically described in **Methods** and **Supplementary Note 2**. An example of the original (green) and the noisy spectrum (blue) corresponding to a pixel of **Supplementary Fig. 4c** with 4.5% superimposed random noise is visualized in **Supplementary Fig. 4d**. **Supplementary Fig. 4e-g** present the recovered maps of the *Eigenfluence* parameters $m_1(\mathbf{r})$, $m_2(\mathbf{r})$ and $m_3(\mathbf{r})$ after inversion and interpolation in the convex hull of the grid. **Supplementary Fig. 4h-j** present the sO_2 estimation using linear unmixing (**h**), eMSOT sO_2 estimation (**i**), as well as the actual simulated sO_2 map (**j**). **Supplementary Fig. 4k** presents the corresponding errors in sO_2 estimation of

eMSOT (blue points) and linear unmixing (red points) in all pixels of the analyzed area, sorted per depth. The sO_2 estimation error maps in the whole analyzed area were used for statistically evaluating the eMSOT performance.

Upon evaluation of the method on a set of 2358 created simulations, we observe that in the physiological range of mean tissue oxygenation between 30% and 80% the mean sO_2 estimation error ranges from 2.4% to 3.4% depending on the levels of random noise, while in ~97% of the cases the sO_2 error did not exceed 10% (**Supplementary Table 1**). We did not observe dramatic performance differences between different mean optical properties or different structures of the optical properties. We further did not observe significant performance degradation with high levels of superimposed noise indicating that the inversion scheme is rather robust to noise. The largest errors were observed in the case of less than 30% mean tissue oxygenation. In this case the mean sO_2 error was 5% and in ~97% of the cases the error was less than 15%. The results of the statistical evaluation of the method over all simulations tested are analytically presented in **Supplementary Table 1**.

Supplementary Fig. 4l presents the mean sO_2 error of linear unmixing and eMSOT corresponding to each simulated data-set tested, while **Supplementary Fig. 4m** present the histogram of the mean sO_2 error corresponding to all simulations. In 88% of all cases tested, eMSOT offered a lower mean estimation error than conventional linear unmixing. In the rest 12% of the cases linear unmixing offered a better estimation, but the mean sO_2 errors were comparable and both were lower than 8%. Finally, **Supplementary Fig. 4n** presents a histogram of the relative sO_2 error yielded by linear unmixing over eMSOT for all simulated data-sets tested and for simulated tissue depths>5 mm; indicating that eMSOT typically offered 3 to 8-fold enhanced sO_2 estimation accuracy in deep tissue.

The statistical evaluation of **Supplementary Table 1** corresponds to the application of a polar grid of an angle step of $\pi/20$ rads and a radial step of 0.14 cm (40 grid points). The effect of the grid density on the sO_2 estimation accuracy was further tested through the application of different grid densities containing 12, 30, 49 and 108 grid points deployed in a $\pi/4$ disk area; the results are summarized in **Supplementary Table 2**. We observed that the sO_2 estimation accuracy does not increase dramatically with an increased grid density due to the smooth spatial variations of light fluence in tissue.

Supplementary Note 4: Validation of eMSOT with tissue mimicking blood phantoms

Blood phantoms with controlled oxygenation levels were created for validating the eMSOT accuracy under experimental conditions where gold standard is available. Different blood sO_2 levels were created by adding different amounts of Sodium Dithionite ($Na_2O_4S_2$)[4], a chemical that allows for efficient deoxygenation of blood. Control experiments indicated that blood solutions in NaCl and intralipid could be stably retained at 100% sO_2 under no $Na_2O_4S_2$ addition and at 0% under 100 mg/g $Na_2O_4S_2$ addition. When $Na_2O_4S_2$ was added at a concentration of 2-4 mg/g, blood solutions were initially deoxygenated but would gradually change to higher oxygenation levels.

A number of cylindrical (diameter 2cm) tissue mimicking solid blood phantoms were created consisting of 3%-5% blood in a solution of NaCl, intralipid (2%) and low temperature melting Agarose. Four different states of background blood oxygenation were formed though the administration of 100 mg/g $Na_2O_4S_2$ (corresponding to 0% sO_2 background), 3 mg/g $Na_2O_4S_2$, 4 mg/g $Na_2O_4S_2$ (corresponding to an unknown and spatially varying sO_2 in background) and 0 mg/g $Na_2O_4S_2$ (corresponding to 100% sO_2 background). A 3mm diameter insertion containing a sealed capillary tube filled with 20% blood at 0% sO_2 and 100% sO_2 was introduced at a depth of 5-8mm within each solid blood phantom. The phantoms were imaged using MSOT and the images were analyzed using the eMSOT method and conventional linear unmixing.

Supplementary Fig. 5a-b present the application of the eMSOT method in the case of a uniform phantom of 0% sO_2 and a phantom of 100% sO_2, respectively. **Supplementary Fig. 5c-d** present the sO_2 estimation error of the eMSOT method (blue dots) and linear unmixing (red dots) for all analyzed pixels sorted per imaging depth.

Supplementary Fig. 5e-f present the application of the eMSOT method in the case of a phantom with an unknown, non-uniform sO_2 background and an insertion of 0% sO_2 blood. The eMSOT grid is placed appropriately to cover the insertion area. **Supplementary Fig. 5g-h** present the initial spectrum in the insertion area [$P(\mathbf{r},\lambda)$] and the sO_2 estimation using linear unmixing (**g**) as well as the corrected spectrum [$P^{\text{eMSOT}}(\mathbf{r},\lambda)$] and sO_2 estimation using eMSOT method (**h**). **Supplementary Fig. 5i** summarizes the sO_2 estimation error of linear unmixing (red) and eMSOT method (blue) corresponding to the insertion area in the case of 8 different blood phantoms (4 different backgrounds and 2 different insertions per background). eMSOT offers higher accuracy with an sO_2 estimation error that is typically less than 10%, as opposed to linear unmixing that can be associated with errors as high as 30%. Finally, **Supplementary Fig. 5i** presents the fitting residual of linear unmixing (red) and eMSOT (blue) in each case.

Supplementary Note 5: Application of eMSOT on experimental tissue images

In experimental tissue data (muscle and tumor analysis) the prior $\acute{m}_1(\mathbf{r})$ and $\acute{m}_3(\mathbf{r})$ maps were computed as described in **Supplementary Note 2** by using a 3D FEM DE light propagation model and 20 sO_2 maps corresponding to 20 consecutive MSOT slices (with a step size 0.5 mm) surrounding the central slice to be analyzed (**Supplementary Fig. 6a**). This was performed in order to provide robust *Eigenfluence* prior estimates even in cases of substantial sO_2 variations in the 3D illuminated volume (MSOT illumination width ~ 1 cm). **Supplementary Fig. 6b** presents the prior $\acute{m}_1(\mathbf{r})$ map corresponding to an animal imaged *post-mortem* after CO_2 breathing.

eMSOT accuracy depends on the quality of the measured optoacoustic spectra in the grid area. For ensuring successful application, an image area of high intensity (high SNR) and fidelity (visually presenting no reconstruction artefacts e.g. due to ill

acoustic coupling) and typically corresponding to the central-upper part of the image (corresponding to the focal area of the ultrasound sensors and eliminating the possibility of reconstruction artefacts due to the limited angle of coverage) was selected for applying the eMSOT method. Upon manual segmentation of an area, a polar grid is automatically applied in the image domain (**Supplementary Fig. 6c**). The grid point location is automatically updated so that the points occupy the highest intensity pixels in their local vicinity. Grid points that correspond to image values under a predefined threshold (i.e. red points in **Supplementary Fig. 6c**) are excluded from the inversion process. The measured optoacoustic spectra corresponding to the grid points are in the following used in the context of the constrained inversion algorithm described in **Methods** and **Supplementary Note 2** to obtain estimates of $m_1(\mathbf{r}_{p,l})$, $m_2(\mathbf{r}_{p,l})$ and $m_3(\mathbf{r}_{p,l})$ for each grid point $\mathbf{r}_{p,l}$. **Supplementary Fig. 2d** presents the prior $\acute{m}_1(\mathbf{r}_{p,l})$ (blue line), the limits of search space (blue vertical lines) and the $m_1(\mathbf{r}_{p,l})$ estimated by the constrained inversion (red line) for a radial line of the grid in **Supplementary Fig. 6c**.

Upon the estimation of $m_1(\mathbf{r}_{p,l})$, $m_2(\mathbf{r}_{p,l})$ and $m_3(\mathbf{r}_{p,l})$ in all grid points, the *Eigenfluence* maps for the intermediate grid points are computed by means of cubic interpolation (see **Methods**). **Supplementary Fig. 6e, f** presents the $m_2(\mathbf{r})$ (**e**) and $m_1(\mathbf{r})$ (**f**) *Eigenfluence* maps corresponding to the same tissue area imaged under different physiological conditions, namely *post-mortem* after CO_2 breathing (left), *in-vivo* under 20% O_2 breathing (middle) and *in-vivo* under 100% O_2 breathing (right). While the $m_2(\mathbf{r})$ spatial map that corresponds mainly to tissue depth remains relatively unchanged under all three physiological conditions, $m_1(\mathbf{r})$ that corresponds more to background tissue oxygenation presents substantial differences between the three different states. The *Eigenfluence* maps are used to correct for the wavelength dependence of light fluence in the selected tissue area (**Methods**) and in the following blood oxygen saturation maps are computed using non-negative constrained least squares fitting of the corrected eMSOT image with the spectra of oxy- and deoxy-hemoglobin (**Supplementary Fig. 6g)**. Pixels that are associated with a fitting residual above a certain threshold are excluded from the sO_2 maps.

After eMSOT application, the raw optoacoustic spectra (blue lines in **Supplementary Fig. 6h** left) are decomposed into the element-wise product of the corrected normalized absorption spectra (blue lines in **Supplementary Fig. 6h** middle) and the estimated light fluence spectra (**Supplementary Fig. 6h** right). While linear fitting with the spectra of oxy- and deoxy-hemoglobin results in a high fitting residual and an inaccurate sO_2 estimation when applied on the raw optoacoustic spectra (red lines in **Supplementary Fig. 6h** left), it results in a low fitting residual after eMSOT correction (red lines **Supplementary Fig. 6h** middle) independently of tissue depth.

Supplementary Note 6: Imaging tumor hypoxia with eMSOT and histological validation

Mice (n=8), bearing orthotopically implanted 4T1 mammary tumors were imaged with MSOT at transverse slices in the lower abdominal area (schematic representation in **Supplementary Fig. 7a**). **Supplementary Fig. 7b** presents an anatomical optoacoustic image showing a slice which corresponds approximately to the central section of the tumor. The tumor region (upper right part of the image) can be recognized as it displays an enhanced contrast and different anatomic characteristics as compared to the symmetric normal tissue region. The tumor region is manually segmented (dashed segmentation line, **Supplementary Fig. 7b**). The eMSOT grid is set to cover the tumor area as well as adjacent healthy tissue (**Supplementary Fig. 7b right**).

After MSOT imaging, the mice were sacrificed and prepared for histological analysis. A subset of the mice (n=4) were examined for tumor and tissue anatomy. Following MSOT acquisition, the mice were frozen and the lower abdominal region containing the tumor mass (dashed lines in **Supplementary Fig. 7c**) was cryosliced in transverse orientation, similar to the one of MSOT imaging (see **Supplementary Fig. 7a**). True color images of the whole body, including the tumor mass, were obtained and histological slices derived thereof were isolated for H&E staining. **Supplementary Fig. 7d-g** presents an anatomical optoacoustic image at the central tumor cross-section (**d**), the corresponding cryoslice true color photography (**e**), H&E tumor staining (**f**) and eMSOT sO_2 analysis (**g**). The cryoslice true color photography displays the tumor heterogeneity, presenting sub-regions with prominent red color (marked in **Supplementary Fig. 7e** with an asterisk). These central necrotic areas, appearing to be suffused with blood, spatially correlate to the central hypoxic region in the core of the tumor as identified in the eMSOT image (**Supplementary Fig. 7g;** marked with an asterisk). Central necrotic areas could be confirmed by H&E staining (**Supplementary Fig. 7f**).

Another subset of the mice (n=4) was examined for functional characterization of the tumors through CD31/Hoeachst33342/Pimodinazole histological staining. Throughout this process, the tumors were excised and the 3D orientation of the tumor with regard to the MSOT image was retained (**Supplementary Fig. 7h,** lower picture). In the following, the excised tumors were sectioned and ~8 µm thick slices were immunohistochemically stained for studying micro-vascularization (CD31 staining) and cellular hypoxia (Pimonidazole staining). Vascular perfusion was determined following Hoechst33342 detection.

Supplementary Fig. 7i presents the eMSOT sO_2 estimation of two tumors presenting different levels of oxygenation. The tumor areas, as identified by the anatomical images, are segmented with a yellow dashed line. The average sO_2 levels of the central tumor areas (blue dashed rectangle) are further displayed in the image. The corresponding CD31 staining, as shown in **Supplementary Fig. 7j** reveals a dense tumor microvasculature in both tumors. This might explain the high tumor contrast in optoacoustic imaging. Hoechst 33342 staining (**Supplementary Fig. 7k**) reveals substantial differences in the perfusion patterns of the two tumors, with the first tumor appearing to be perfused both in the boundary (grey dashed box) and the core (green dashed box). In an effort to quantify the perfusion patterns, the ratio of the Hoechst image intensity in the core vs the boundary

was computed (intensity ratio 48%). The second tumor displays less perfusion in the core, as compared to the boundary (intensity ratio 19%). This finding indicates less functionality of the microvasculature in the core, which might explain the lower eMSOT sO_2 values as compared to the first tumor. The less perfused tumor areas (dark areas in **k**) appear spatial congruence with the areas of reduced blood oxygenation revealed by eMSOT (**i**). The non-perfused tumor areas further appear spatially correlated to cell hypoxia as identified by Pimonidazole staining (**l**, green). Cell hypoxia, as determined by Pimonidazole staining, may be a consequence of both, perfusion hypoxia (revealed by Hoechst33342 and eMSOT) and also diffusion hypoxia, which does not display eMSOT signal. Although, due to technical reasons, it may be challenging to achieve exact co-registration between *in-vivo* eMSOT tumor images and *ex-vivo* histology, the given histological analyses present strong evidence on the ability of eMSOT to detect perfusion related hypoxia within solid tumors. Furthermore, clear discrimination of different levels of hypoxia within single tumors, as well as intra-tumoral hypoxia-related heterogeneity could be demonstrated.

Supplementary References

1 Mohajerani, P. *Robust Methods for Fluorescence Imaging and Tomography* Doctoral Dissertation thesis, TU München, (2014).

2 Jacques, S. L. Optical properties of biological tissues: a review. *Physics in medicine and biology* **58**, R37 (2013).

3 Wang, L., Jacques, S. L. & Zheng, L. MCML—Monte Carlo modeling of light transport in multi-layered tissues. *Computer methods and programs in biomedicine* **47**, 131-146 (1995).

4 Briely-Sabo, K. & Bjornerud, A. in *Proc. Intl. Sot. Mag. Reson. Med.* 2025.
